# A thorough insight into the life cycle of the Epstein-Barr virus. From the molecular to the organismal level

**DOI:** 10.1016/j.crmicr.2025.100505

**Published:** 2025-11-03

**Authors:** Andrii Zaremba, Polina Zaremba, Svіtlana Zahorodnia

**Affiliations:** D. K. Zabolotny Institute of Microbiology and Virology of NASU, 154 Acad. Zabolotny Str., Kyiv, 03143, Ukraine

**Keywords:** EBV, Immune system, Latency program, Germinal center theory, Reactivation, Virion assembly

## Abstract

Viruses are known to be a specific form of life capable of reproduction exclusively by subjugating cellular life forms, where they have reached impressive heights. Especially complex and therefore interesting are pathogens, which have chosen maximum adaptation to the host as their survival strategy.

In this review, we attempt to comprehensively describe the life cycle of one of the most adapted human pathogens – the Epstein-Barr virus (EBV). Despite significant efforts to study the biology of this virus, forming a complete picture of its life cycle based on scattered reports is currently a difficult task. This makes the understanding of EBV fragmentary and often insufficient for an analysis of the experimental data. Based on the currently available non-contradictory data, we consider most stages of the EBV life cycle and interpret them in the context of its survival strategy. We emphasize the features of EBV tropism to different types of susceptible cells, and describe the stages of entry and initial establishment of infection, the transition to latency and its various stages in the context of gentle subjugation of the host metabolism, the use of the host immune system for maximum disguise, the mechanisms of reactivation, and the lytic cycle and the associated remodeling of cell membranes.

## Highlights

• Establishment of Epstein-Barr virus infection is accompanied by the involvement of the host immune system in the regulation and self-limitation of the infectious process.
• EBV is able to switch host cell types according to the purpose: transmission or survival.
• EBV delicately manipulates the activity of host genes to avoid unwanted death and establish lifelong latent infection.
• EBV reactivation in resting memory B-cells is dependent on natural signaling pathways that accompany the normal activation process.

## Graphical abstract

**Figure:**
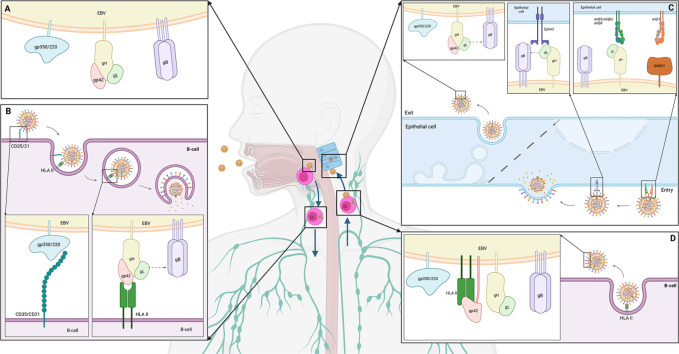

## 1 Introduction

Epstein-Barr virus (EBV), also known as human herpesvirus 4, is currently classified in the family Herpesviridae, subfamily Gammaherpesvirinae, and genus Lymphocryptovirus (Schoch et al., 2020). EBV was identified in 1964 in a study of Burkitt's lymphoma, historically it is the first clear link between an infectious disease and human cancer. It was later found to be associated with other types of lymphoma, including Hodgkin lymphoma, post-transplant lymphoproliferative disorders (PTLD), non-Hodgkin lymphoma in HIV-infected individuals, T-cell lymphoma, and NK/T-cell lymphoma (Yu and Robertson, 2023; Xiao et al., 2025). EBV has also been associated with nasopharyngeal carcinoma and a subtype of gastric cancer. In addition to its association with various human cancers, the virus is also the cause of infectious mononucleosis in primary infection and oral hairy leukoplakia in immunocompromised individuals. This pathogen and, consequently, its infection are also considered as one of the factors directly influencing the pathogenesis of autoimmune diseases such as systemic lupus erythematosus and multiple sclerosis (Zhao et al., 2024).

More than 90 % of the world's population are asymptomatic EBV carriers, making it one of the most successful human pathogens (Wong et al., 2022). This is also evidenced by the data of phylogenetic analysis, which postulates 12 million years of coevolution of EBV with humans (Ehlers et al., 2010). The widespread prevalence of this virus is largely due to its predominant tropism for human B-cells, which is combined with the ability of all herpesviruses to exert extensive control over the metabolism of the infected cell, as well as the presence of two phases of the life cycle: lytic and latent. Thus, a pool of lymphocytes with a memory B-cell phenotype and the virus genome in episomal form remains in the EBV-positive organism.

Despite the truly widespread distribution and clear causal relationship between EBV and various diseases, including cancer, there is currently only a small number of works devoted to the analysis of the life cycle of this virus. That is unfortunate, considering its capability of an exceptional level of control of the metabolism and behavior of an infected cell. Available reviews mostly focus on individual aspects of either the EBV life cycle or its interaction with the host organism. That, of course, allows us to generally understand the life strategy of this virus. However, at the same time it leads to a one-sided and often incomplete interpretation of experimentally obtained data, which can and probably lead to false hypotheses, the refutation of which requires time and resources.

In this review, we attempt to provide a comprehensive picture of the EBV life cycle. Recognizing that a detailed overview of the EBV infection in both B-cells and pharyngeal epithelial cells is beyond the scope of a single review, we have focused on a sequential and detailed overview of the B-cell infection. At the molecular level we consider events that accompany the establishment of the long-term latent infection. We show the key role of the host immune system in this process and how the amazingly complex interaction with the host allows the virus to self-limit itself and fine-tune its long-term survival. In addition, we describe the processes of EBV reactivation and the subsequent stepwise initiation of the lytic stage of its life cycle. Although it should be noted that the creation of a complete picture of pathogenesis in the case of restriction to only one cell type is impossible, we therefore touch on some critical aspects of epithelial EBV infection as well.

During the preparation of the review, we used the online resource PubMed and its tools. We did not restrict the date or type of publications, however, in the case of newer data that reasonably refuted previously known results, we relied on the most recent ones. In the absence of knowledge on a specific process of the EBV life cycle, we depended on data obtained for other herpesviruses based on their phylogenetic affinity. Preprints were not considered as reliable data.

## 2 Virion and genome structure

**Fig. 1:**
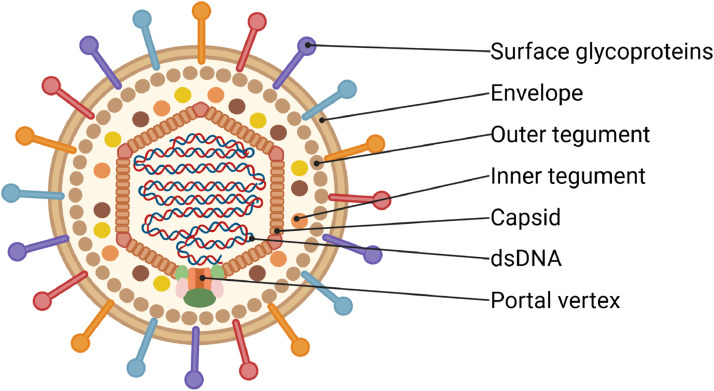
The structure of the EBV virion. Created in BioRender. Zaremba, P. (2025) https://BioRender.com/ pe26pi4.

EBV generally has a viral particle structure similar to other herpesviruses (Fig. 1). In particular, it is a complex virus with a virion diameter of about 125 nm (Draganova et al., 2021) and a three-layer structure, which includes a surface lipid bilayer with integrated viral glycoproteins responsible for tropism and membrane fusion, as well as host cell surface proteins; an internal pseudo icosahedral nucleocapsid; and a pleomorphic tegument located between them, which consists of 20–40 different proteins (Fig. 1). The main viral glycoproteins are represented by five proteins: gp350/220, gp42, BMRF2, gH/gL, and gB. gH/gL and gB form a highly conserved among all herpesviruses fusion core, which is responsible for the fusion of the lipid membranes of a cell and the virus (Möhl et al., 2016). The nucleocapsid consists of the major and minor capsid proteins, combined into 150 hexamers and 11 pentamers (*T* = 16), as well as the minor proteins Tri1 and Tri2. The latter form triplexes (Tri1+Tri2A+Tri2B) and are located in the gaps between the major pentamers and hexamers, where the former are located at all capsid vertices except the one with the portal protein. Between the inner nucleocapsid and the outer lipid bilayer membrane is the tegument (Möhl et al., 2016) and the capsid-associated tegument complex (CATC) (Draganova et al., 2021; Li et al., 2020).

**Fig. 2:**
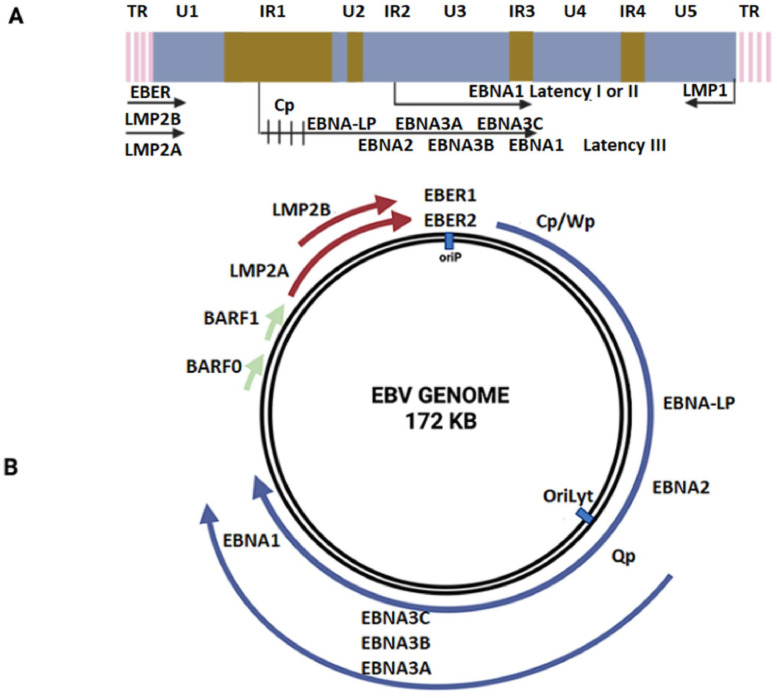
Epstein–Barr virus genome organization. (A) EBV lineal genome. (B) EBV circular genome representation (Osorio et al., 2022).

The inner part of the capsid contains linear dsDNA of 170–180 kb in size, which encodes from 85 to over 100 viral proteins and 44 miRNAs (Židovec Lepej et al., 2020; Smatti et al., 2018). In its linear form, viral dsDNA is characterized by the presence of two complementary regular regions at the ends of a 538 bp duplex (namely, terminal repeats or TR). Later, during the establishment of EBV infection, pairing of these regions ensures successful ligation of the ends of dsDNA and, accordingly, the formation of a circular (episomal) form of the genome (Fig. 2). In addition to terminal repeats, the EBV genome is characterized by the presence of four internal repetitive regions (IR1, IR2, IR3 and IR4), which have no homology with the TR and encode some of the viral factors. The number of repeats themselves is a specific feature and depends on the particular isolate. The transcriptional features of these regions, including the exact mechanisms of alternative splicing, still remain poorly studied (Ba Abdullah et al., 2017). However, previous studies link them to the transforming ability of the Epstein-Barr virus (Tierney et al., 2011). Terminal and internal repeats divide the EBV genome into 5 non-repeating parts U1-U5, which also encode viral factors. In general, the features of the structure, diversity, regulation of the EBV genome, as well as alternative splicing, are an open question that is actively being studied (Wongwiwat et al., 2022; Borozan et al., 2018; Agwati et al., 2022; Houldcroft, 2019).

## 3 Features of the first stages of the Epstein-Barr virus interaction with B-cells and pharyngeal epithelial cells. Dependence of tropism on the place of reproduction

Most often, primary infection with this herpesvirus occurs in childhood and is asymptomatic. Children are exposed to EBV often in the first year of life when interacting with their parents or other relatives. The average age of primary infection is directly correlated with the economic development of the country: the first contact occurs later in more developed countries (Gerpe et al., 2020). If the infection occurred in adolescence or in adulthood, the likelihood of the aforementioned infectious mononucleosis, i.e. an acute immune reaction to EBV replication, is significantly increased.

The EBV transmission is mainly carried out through contact of a susceptible organism with the fluids of an infected one. Infectious mononucleosis is even called "the kissing disease" because most often it is the saliva that contains the virus in sufficient concentration for further infection. Accordingly, the canonical entrance gateway for EBV is the pharynx. Currently, the dominant theory is that the primary cells that are infected in this case are the pharyngeal epithelium. This is due to the high tropism of the virus to these cells, as well as its obvious and logical connection between the gateway of EBV and the cell localization. However, there is another theory, which also has a number of confirmations. In its case, it is assumed that EBV virions with the flow of saliva enter the palatine tonsils and their crypts (Yu and Robertson, 2023). There they cross the thin layer of epithelium and directly infect naive B-lymphocytes. In this case, the infection of epithelial cells is considered a secondary phenomenon. This is confirmed by the 2015 cohort study, which examined blood and oral wash samples from a total of 40 young adults (Dunmire et al., 2015). It was found that in peripheral blood, a significant increase in viral DNA was observed 1–2 weeks before the onset of symptoms of infectious mononucleosis and the increase in viral load in saliva. This, in addition to the generally accepted theory regarding the entrance gateway, is also inconsistent with the generally accepted theory of an exclusively lytic cycle of EBV inherent in epithelial cells and a predominantly hidden (latent) infection with only periodic reactivation inherent in B-cells. The third option is not excluded, in which EBV reproduction in the early stages of infection occurs equally in both lymphoid and epithelial tissue of the pharynx (Hadinoto et al., 2008). However, it is obvious that the interaction of the Epstein-Barr virus with the human body is more complex than previously thought, differs in the initial and late stages of infection, and also requires additional and thorough research in this direction.

**Fig. 3:**
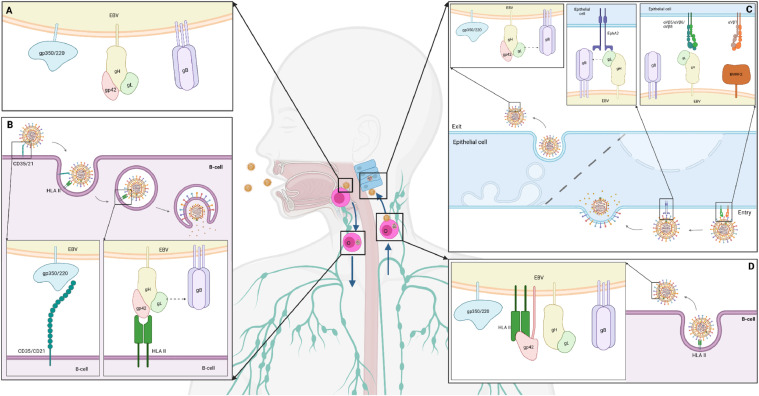
The general scheme of EBV entry into B-lymphocytes and epithelial cells during infection. (A) Composition of EBV surface glycoproteins during the first contact. (B) Virus entry into a B-cell. (C) Exit of virions from a B-cell and composition of their surface glycoproteins. (D) Entry and exit of viral particles to/from an epithelial cell and composition of the surface glycoproteins. Created in BioRender. Zaremba, P. (2025) https://BioRender.com/ho2l0b9.

Nevertheless, the presence of a clearly expressed tropism of EBV specifically to pharyngeal epithelial cells and B-lymphocytes is a proven fact. At the molecular level, the process of entry and reproduction of this virus differs significantly between these two cell types (Jean-Pierre et al., 2021). In particular, in the case of epithelium, it is classically believed that the interaction of EBV with a host cell begins with the binding of the surface glycoprotein BMRF2 to the most common integrins (αVβ1 and others; Fig.3, C) (Xiao et al., 2008; Anderson et al., 2014). In this case, a specific integrin-binding structural motif of 3 amino acids (arginine, glycine and aspartate or RGD) in the composition of BMRF2 is responsible for the interaction with the ligand. A similar motif (KGD) is present in the composition of gH. This glycoprotein, as part of the gH/gL complex, is capable of additionally recognizing integrins αVβ5, αVβ6, and αVβ8 (Chesnokova and Hutt-Fletcher, 2011), which provides a higher level of stabilization of the virus-cell interaction. At this stage, only gB (class III fusion protein) is missing for the virus to enter the cytoplasm in the zone of maximum proximity of the cellular and viral lipid membranes (Backovic et al., 2009). According to recent data, the recruitment of this factor is provided by the ephrin receptor with tyrosine kinase activity EphA2, which is one of the surface signaling ligand proteins. The ectodomain of this protein consists of four regions: a ligand-binding domain that interacts with gH/gL and gB, a cysteine-rich region, and 2 fibronectin-like repeats that also bind to gB. Accordingly, EphA2 can be considered as a bridge that provides proximity of gB to gH/gL. The latter are regulators of gB functioning and stimulate its conformational rearrangements, which ultimately lead to the release and incorporation of the WY 112–113 and WLIW 193–196 fusion loops into the cell membrane and virus entry. The significant role of EphA2 at this stage of the life cycle is evidenced by recent data on a 90 % decrease in the efficiency of EBV entry into the cell in its absence (Chen et al., 2018). However, its overexpression results in the opposite effect (Chen and Longnecker, 2019), although the exact molecular mechanisms of EphA2-mediated interaction of gB with gH/gL are unknown.

In the case of B-lymphocytes, the attachment receptor is the glycoprotein gp350/220, which is more common in the EBV envelope (Fig. 3, B). It is represented in two isoforms of 350 kDa and 220 kDa, which are the result of alternative splicing of a single gene and perform a generally identical function. Namely, through molecular mimicry with complement components, they recognize either complement receptor 1 (CR1/CD35) or complement receptor 2 (CR2/CD21) (Joyce et al., 2025). After the initial interaction between gp350/220 and the CR1/2, endocytosis of the immobilized virion occurs. Although there is still no understanding of this process mechanisms at the molecular level, studies using chlorpromazine (an inhibitor of clathrin-mediated endocytosis) and electron microscopy data confirm the presence of a clear cause-and-effect relationship between the formation of the gp350/220-CR1/CR2 complex and EBV endocytosis (Valencia and Hutt-Fletcher, 2012). After internalization of the viral particle, the C-terminal domain of gp42 specifically interacts with the β-chains of HLA class II, which are widely represented on the surface of B-cells as antigen-presenting factors of immunity. Actually, gp42 is a part of the gH/gL/gp42 complex, where the main structural unit is gH, which is permanently linked to gL through its N-terminal domain. The remaining three gH domains, in turn, bind to gp42, where the interaction with the d-II domain of gH and its KGD motif is considered key (Möhl et al., 2019). Binding of gp42 as part of the heterotrimeric complex with HLA class II triggers a series of conformational rearrangements that ultimately lead to the interaction of gL with gB through residues Q54 and K94. This, in turn, causes conformational rearrangements of this fusion factor similar to those described above and leads to the fusion of the viral and endosomal membranes.

It was clearly noticeable that gH is capable of interacting with both gp42 (a factor necessary for successful infection of B-cells) and with integrins of epithelial cells. In both cases, the KGD motif is key. Thus, gH is capable of competitively recognizing either gp42 or αVβ5/αVβ6/αVβ8. Accordingly, in the case of a sufficient amount of gp42 in the composition of the virion membrane, most of the KGD motifs of gH are inaccessible for interaction with integrins of epithelial cells, i.e., the tropism of such virions to epithelial cells will be reduced. In the opposite case, the tropism will be reduced to B-lymphocytes. Thus, the EBV progeny from B-cells will have a relatively higher affinity for pharyngeal epithelial cells, since most of the gp42 will be immobilized by HLA class II. Conversely, the EBV progeny from epithelial cells will be more related to B-lymphocytes, since gp42 will be present in sufficient quantities on the surface of virions, and the KGD motif of gH will be unavailable for interaction with integrins (Fig. 3) (T. Murata, 2023; Möhl et al., 2019; Sathiyamoorthy et al., 2016).

The above also indicates in favor of infection of B-lymphocytes in the case of primary EBV entry into the human body.

## 4 The earliest cytoplasmic and nuclear events after the entry. Pre-latent phase

The next step after entry for EBV is the transport of the nucleocapsid to the nuclear membrane with subsequent entry of the viral genome into the cell nucleus. It is currently believed that this process is identical for both types of susceptible cells, although the precise mechanisms have not yet been described. However, based on data on more studied members of the Herpesviridae family, homology in the structure of tegument proteins and available studies of knockout of their genes in the EBV genome, it is generally possible to understand the mechanism of EBV entry into the nucleus of susceptible cells (Li et al., 2020).

**Fig. 4:**
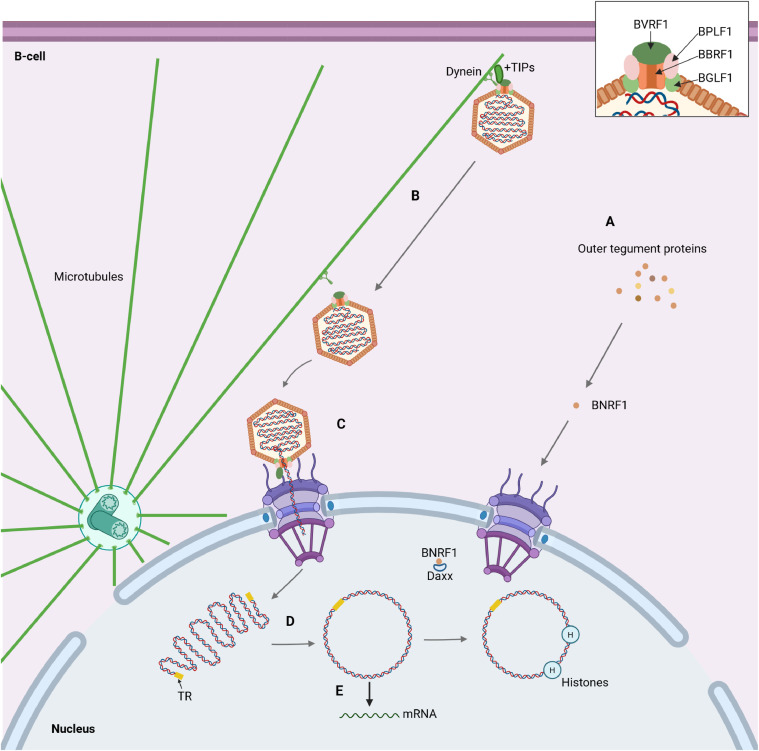
Subsequent events after EBV enters a B-cell. (A) Dissociation of outer tegument proteins; (B) retrograde transport of the EBV nucleocapsid; (C) entry of the viral genome into the nucleus; (D) cyclization of the EBV DNA and interaction with histones; (E) initial synthesis of viral proteins. Created in BioRender. Zaremba, P. (2025) https://BioRender.com/zpneeqe.

First of all, after uncoated EBV enters the cytosol the dissociation of the outer tegument proteins occurs (Fig. 4, A). The latter differ from the inner tegument proteins in their interaction mainly with the virus envelope and in their lower compaction. Immediately after infection, they function as the primary effectors on the host metabolism by reducing cell protein synthesis and counteracting the stimulation of apoptosis. For example, BNRF1 interacts with the DAXX protein, thereby disrupting the formation of the DAXX-ATRX complex, which leads to inhibition of DNA methylation and, accordingly, allows to preserve the transcriptional activity of the EBV genome. In addition, most of the outer tegument proteins are constitutively synthesized during the infection cycle (especially the lytic stage), which suggests their important role in the EBV reproduction. The inner tegument proteins (especially BPLF1) interact with dyneins, as well as with microtubule plus-end-associated proteins (+TIPs), including dynactin (Fig. 4, B) (Döhner et al., 2021). After association with the cellular cytoskeleton, the EBV nucleocapsid, like other herpesviruses, is retrogradely transported to the centrosome, which is mostly located near the cell nucleus, at a speed of about 1–2 μm/s. Thus, EBV accumulates near the nuclear membrane and its further transport is probably similar to that of HSV-1. It has been proven that HSV-1 can move between the centrosome and the nucleus due to the presence of a nuclear localization signal in the pUL36 component, which, in turn, is a BPLF1 homolog (Döhner et al., 2021).

BPLF1 probably also mediates the primary interaction with the nuclear pore complex (NPC), since pUL36 provides the primary interaction of the HSV-1/2 nucleocapsid with the long cytoplasmic filaments of the NPC formed by the nucleoporin Nup358 (Fig. 4, С). After this, BVRF1 (another CATC protein) interacts with additional NPC components: the nucleoporins Nup214 and hCG1, which are located at the base of Nup358, closer to the nuclear membrane. The same protein at the apex occupied by the portal dodecameric complex of the BBRF1 forms the so-called cap, which leaves its position, probably due to interaction with nucleoporins of the NPC base. Thus, the portal complex, which forms a channel with a diameter of 36 Å between the interior of the capsid and the external environment, is open. The further mechanism of the release of linear DNA from the capsid and its entry into the nucleus is now considered to be very alike to that in bacteriophages based on structural similarity and involves mechanical exit of the genome through the portal due to excessive intracapsid pressure (Brandariz-Nuñez et al., 2019).

After complete entry into the nucleus, the viral genome is converted to an episomal form (Fig. 4, D). This process has also not been fully studied for EBV. However, based on the similar organization of genomes within the entire Herpesviridae family, it can be assumed that the EBV genome in the cell nucleus is cyclized by the formation of duplex regions between the head and tail inverted terminal repeats. After that, due to the involvement of the cellular machinery of homologous recombination, a covalent connection of both strands of the DNA duplex occurs (Strang and Stow, 2005; López-Muñoz et al., 2021), probably with the formation of a Holliday cross-shaped junction (Pérez-Losada et al., 2015).

In parallel with cyclization, the viral genome interacts with histones. Characteristically, because EBV-infected cells are mostly in G0-phase, the classical histone H3, which is synthesized only in the S-phase of the cell cycle, is replaced by histone H3.3, which is produced more evenly (Clatterbuck Soper and Meltzer, 2023). This same histone forms the tripartite complex ATRX/DAXX/H3.3, where ATRX is a chromatin remodeling factor, and DAXX functions as a chaperone for the correct formation of the H3.3/H4 complex (Elsässer et al., 2012). This multicomponent nucleosome complex then recruits the methyltransferase SETDB1 (Voon and Wong, 2016). This leads to K9me3 on H3.3 and, consequently, suppression of transcriptional activity of the corresponding DNA regions. In the case of EBV, this process is inhibited by the aforementioned component of the outer tegument, the BNRF1 protein, which immobilizes DAXX. Given that the EBV genome is unmethylated in the first period after entry, within 1–2 weeks after infection there is a disordered synthesis of various factors of the virus life cycle, both lytic and latent (Fig. 4, E) (Inagaki et al., 2020; Jochum et al., 2012; Kalla et al., 2010; Murata et al., 2014). This period is called the pre-latent abortive lytic phase or simply the pre-latent phase. It is believed to be essential for the survival of the infected cell, as it is during this period that components of the lytic cycle are synthesized, such as BALF1 and BHRF1 (anti-apoptotic BCL2-like proteins), and BNLF2A, BGLF5, BILF1, BDLF3 (inhibitors of HLA expression) (Quinn et al., 2016; Altmann and Hammerschmidt, 2006).

## 5 Establishment of infection. Transcriptional latency program IIb

**Fig. 5:**
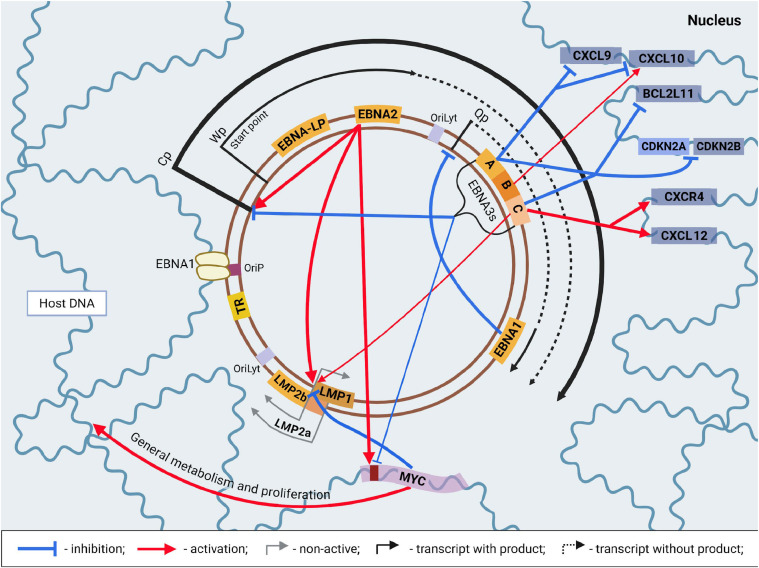
The general scheme of the EBV episome and the pattern of mutual influence of the pathogen and the host cell characteristic of the transcriptional latency program IIb. The thickness of the arrows corresponds to the relative intensity of the influence. Created in BioRender. Zaremba, P. (2025) https://BioRender.com/j42e1la.

Coordinated transcription of the EBV genome begins with the activation of the Wp promoter cluster, which forms IR1 and controls the transcription of the EBNA2 and EBNA-LP gene clusters (Fig. 5). The result is the production of a single bicistronic mRNA (Elliott et al., 2004), which is further processed and translated into mature EBNA2 and EBNA-LP. Although Wp has the potential to transcribe the entire EBNA locus, it is currently believed that the movement of the transcription machinery is relatively inefficient when initiated from Wp. Accordingly, this promoter can be considered weak (Price and Luftig, 2014). EBNA2 and EBNA-LP are functionally closely related and are considered transcriptional coactivators that largely jointly influence the transcription of cellular and viral genes (Kang and Kieff, 2015). The mechanism of their action is based on the inhibition of transcription repressors with the simultaneous involvement of transcription activators. Despite the fact that EBNA2 and EBNA-LP are even capable of complex formation, the spectrum of their activities according to current data is somewhat different (Zhao, 2023). In particular, EBNA2 interacts with a transcription factor RBPJ. The latter is a participant in Notch-signaling and ensures repression of target genes after activation of the Notch receptor (Friedrich et al., 2022). According to some studies, EBNA2 also interacts with factors of the ETS, NF-κB and RUNX3 families (Zhao et al., 2011). Other activities of EBNA2 are very similar to those of cellular transcriptional activators and involve the recruitment of coactivators EP300/CBP, chromatin remodeling factors SNF/SWI, and basal transcription factors TFIIH, TFIIB, and TFIIE. In contrast, EBNA-LP as a transcriptional regulator has only the ability to immobilize cellular transcriptional repressors. Although their list is somewhat different, it has been confirmed to include NCOR and HDAC4. In total, host DNA contains thousands of interaction sites for EBNA2 and EBNA-LP (Portal et al., 2013; Lu et al., 2016). Among them ∼33 % of EBNA-LP binding sites are located on promoters. At the same time, only ∼14 % of EBNA2 interaction sites are located in promoter regions (Zhao, 2023). Thus, in total, EBNA2 and EBNA-LP function as modulators of each other's activity within the infected cell. However, only general data on the transcriptional influence of these factors are available.

One of the best studied is the mechanism of transcription activation of the MYC proto-oncogene (Zhao et al., 2011). EBNA2 is predicted to interact (probably through certain cellular transcription factors and in complex with EBNA-LP) with an enhancer ∼556–428 kb upstream of the MYC gene (Pomerantz et al., 2009). Further involvement of other cellular factors allows this enhancer to be brought closer to the MYC promoter region, thus forming a loop-like structure. It should be noted that MYC is an extremely powerful transcription factor that directly or indirectly enhances the transcription of thousands of cellular genes. The latter are often positive regulators of the cell cycle. Disruption of the functioning of this oncogene is one of the most common markers of neoplastic cell degeneration (Dhanasekaran et al., 2022). It is believed that, on a more global level, the period of EBNA2- and EBNA-LP-stimulated MYC accumulation during EBV infection allows the most powerful and rapid escape of the infected cell from apoptosis (Yin et al., 2019). This is especially important in the early stages of the viral life cycle, during a period when its own more precise cell cycle regulators, such as surface antigens, have not yet developed in sufficient quantities.

The effect of EBNA2 and EBNA-LP as a coactivator is not limited to the cellular genome. EBNA2 is a strong stimulator of the Cp promoter, which is located upstream of Wp and, unlike latter, ensures efficient expression of all EBNAs within a single polycistronic transcript (including EBNA2 and EBNA-LP), which is converted into native mRNAs of each of the listed factors by alternative splicing (Saha and Robertson, 2013). This occurs approximately 1–3 days after infection (Alfieri et al., 1991), coincides with peak values ​​of lytic proteins such as BHRF1 and BALF1 (potent inhibitors of apoptosis), and is accompanied by a period of extremely rapid proliferative activity (mitosis every ∼8 h) (Nikitin et al., 2010). However, the direct role of such a switch is still unclear, as in vitro B-cell transformation can occur without the presence of Cp (Mabuchi et al., 2021). It is likely that the in vivo role of this process is significant. Given the simultaneous synthesis of a certain number of lytic genes capable of counteracting premature cell death, which in this case may be caused by MYC-mediated hyperproliferation, a gradual transition of the transcriptional activity of EBV from a strong stimulation of division to a more moderate and orderly one seems likely. For this reason, it is highly probable that switching the expression control from Wp to Cp is necessary. The transcription program of the viral genome with Cp as a promoter and EBNA1, EBNA2, EBNA-LP and EBNA3s as products is called the latency program IIb (Klein et al., 2013).

Currently, based on significant similarity in the region of 90–320 bp of the sequence, it is believed that EBNA3A, EBNA3B and EBNA3C arose as a result of a series of sequential duplications that occurred during the evolution of EBV (Saha and Robertson, 2013). Interestingly, this homologous domain in the case of each of EBNA3 is responsible for the interaction with the transcription factor RBPJ, that was mentioned earlier in the context of EBNA2 (Kalchschmidt et al., 2016). Currently, the most common hypothesis is the presence of antagonistic relationships between EBNA2 and the EBNA3 family, at least due to their competition for RBPJ. This is also confirmed by the fact that factors of this family are able to suppress the stimulation of the LMP2A and Cp promoters caused by EBNA2 (Johannsen et al., 1996). Direct antagonism to EBNA2 allows to gradually weaken the excessive synthesis of MYC according to the degree of EBNA3 accumulation, and divert the infected cell from apoptosis caused by excessively long-term high proliferative activity, dsDNA breaks and, accordingly, activation of the DDR system (DNA-damage response) (Hoffman and Liebermann, 2008). The powerful pro-apoptotic potential of MYC itself should also be taken into account (Harrington et al., 2021). However, the interaction of these viral proteins is far from limited to RBPJ, but, like EBNA2, encompasses a significant part of the genome of both the host and the virus (Styles et al., 2018).

Conditionally, based on the features of functioning as transcription factors, members of the EBNA3 family can be divided into EBNA3A and EBNA3C, which are believed to have similar functionality and are direct agonists, as well as EBNA3B, which is their functional antagonist. Thus, EBNA3A and EBNA3C are capable of suppressing the accumulation of proteins p16^INK4a^ and p14^ARF^, which are alternative transcripts of the CDKN2A gene (Maruo et al., 2006, Maruo et al., 2011; Popov and Gil, 2010). p16^INK4a^ is an inhibitor of the cyclin-dependent kinases CDK4 and CDK6 and, normally, acts as a negative regulator of the cell cycle. p14^ARF^ is an inhibitor of the MDM2 factor, which prevents p53 from accumulating excessively in the cell and, thus, stimulating apoptosis. However, the suppressive effect of EBNA3A and EBNA3C on CDKN2A transcription is not the same as EBNA3C is a better inhibitor (Maruo et al., 2011). In addition to CDKN2A, these factors also affect the transcription of the neighboring gene CDKN2B, which encodes the factor p15^INK4b^. The latter is a functional homolog of p16^INK4a^ and is responsible for similar inhibition of CDK4 and CDK6 (Popov and Gil, 2010). It is currently believed that the effect of EBNA3A and EBNA3C in this case is realized through CtBP-mediated (a family of transcription suppressor factors) chromatin remodeling with the addition of repressive histone marks to the CDKN2A and CDKN2B loci (Skalska et al., 2010). CtBP, in this case, ensures the recruitment of the enzymatic apparatus necessary for this process, and factors EBNA3A and EBNA3C probably directly recognize the target sequences (Jiang et al., 2014).

In addition to their clear deregulatory effects on the control of proliferative activity, EBNA3A and EBNA3C also inhibit the accumulation of the pro-apoptotic BCL-2 family factor BIM (Anderton et al., 2008). BIM, along with other pro-apoptotic BCL-2 s, directly correlates with MYC levels, limiting normally MYC-stimulated proliferation. This is particularly important for the elimination of autoreactive lymphocytes (Enders et al., 2003). As in the case of epigenetic suppression of INK4 family proteins, transcriptional repression of BCL2L11 (the gene encoding BCL-2) occurs through the recruitment of additional chromatin remodeling factors, namely the proteins SUZ12 and EZH2, which are components of the so-called polycomb system and directly of the PRC2 complex (Paschos et al., 2012). The latter, in turn, has methyltransferase activity (EZH2 is the transferase) and introduces repressive marks to the histones of the recruitment locus.

In addition to transcriptional suppression, EBNA3C is also capable of activating transcription. Of all EBNA3s, it is the only factor with such capability. In particular, together with EBNA2, it is a coactivator of the LMP-1 promoter (Lin et al., 2002). EBNA3C also activates the expression of the CXCR4 and CXCL12 genes (Maruo et al., 2011). These factors belong to the family of chemokines and their receptors. Specifically, CXCL12 is a chemoattractant for all cells that strongly express CXCR4, such as B-lymphocytes. The binding of CXCR4 to CXCL12 triggers a G-protein-mediated signaling cascade of a proliferation stimulation (Shi et al., 2020). EBNA3A, through direct binding to intergenic enhancers, inhibits the accumulation of CXCL10 and CXCL9 (two of the three IFN-γ-stimulated chemokines) (Harth-Hertle et al., 2013). In addition to the ability to activate EBNA3C transcription, this also suggests that EBNA3A and EBNA3C somewhat differ in their effects on cellular metabolism, despite a significant level of synergy in the functionality. This is also supported by the data that only about 50 % of the interaction sites of EBNA3A with DNA coincide with those of EBNA3C (Schmidt et al., 2015). Although, as you can see, these identical for both areas are the best studied, as well as their effect on proliferation.

However, the most interesting thing concerns the EBNA3B factor, which, compared to EBNA3A and EBNA3C, has anti-proliferative activity (White et al., 2012). It is actually the least studied of all EBNA3s, which is probably due to data on the lack of necessity of this factor for effective immortalization of B-lymphocytes in vitro, which were obtained back in 1992 (Longnecker et al., 1992). It is now known that in vivo infection with EBNA3B-deficient EBV resulted in much more severe disorders associated with the proliferative activity of B-cells than wild-type EBV (White et al., 2012). This is most likely due to the EBNA3B-mediated stimulation of CXCL10 expression (White et al., 2010). The latter is confirmed by the relatively low recruitment of T-cells to the foci of proliferation of B-cells infected with the defective virus.

Thus, the EBNA3 protein family, during the establishment of the IIb latency program, primarily plays a role in protecting the newly infected cell from MYC-stimulated apoptosis directly and by high proliferation indirectly. At the current level of EBV research, we understand that the mechanism of EBNA3 action is based on significant inhibition of EBNA2 functionality with simultaneous deregulation of the cell's proliferative activity control system. The first is a consequence of direct competition for RBPJ and leads to a gradual decrease in the concentration of MYC. While the second occurs due to a complex process that includes inhibition of the synthesis of the INK4 family proteins (suppressors of cyclin-dependent kinases), stimulation of chemokines CXCR4 and CXCL12 (positive autocrine growth regulation) and inhibition of pro-apoptotic factors p14^ARF^ and BIM. Thus, in parallel with the inhibition of the initial MYC-mediated stimulation of division, there is a replacement and more subtle stimulation of the proliferative activity of the cell with a parallel suppression of its response to pro-apoptotic stimuli such as DNA damage. In addition, at the stage of EBNA3 proteins accumulation, regulation begins to be carried out at a higher level: EBNA3C and EBNA3B as antagonists precisely regulate the release of chemokines CXCL10 and CXCL9, which allows to reduce the detection of infected cells by the host immune system, but at the same time leave them under sufficient immune control. The latter is obviously necessary to reduce the likelihood of uncontrolled division of the pool of infected lymphocytes with a subsequent threat to the life of the host and, accordingly, the spread of the virus.

The last, but not least factor of the EBNA family is EBNA1 (Wilson et al., 2018). In the case of latency program IIb, as well as III (to be discussed later), this protein is produced from a single polycistronic transcript regulated by Cp like the rest of EBNAs. Although in addition to this promoter, EBNA1 is also regulated by a more specific constitutively active Q promoter (Tao et al., 1998). The constitutive activity of the latter is ensured by its interaction with the cellular transcription factor Sp1, which is responsible for a large number of so-called "housekeeping proteins", i.e. proteins necessary for the basic functioning of the cell (Bulfone-Paus et al., 1995). In this case, the activity of Qp is negatively autoregulated due to the binding of EBNA1 to viral DNA 10 nucleotides downstream in the sequence (Westhoff Smith and Sugden, 2013). Thus, during the period of high Cp activity and, accordingly, a significant concentration of EBNA1, the Q promoter is suppressed enough to argue about its lack of activity (Schaefer et al., 1997).

It is currently believed that the main function of EBNA1 within the EBV life cycle is to ensure replication of the viral genome synchronously with host DNA (Niller and Minarovits, 2012). This is achieved due to the specific structure of this protein. Most conventionally, EBNA1 can be divided into N-terminal and C-terminal regions, where the first is responsible for interaction with host DNA, and the second for interaction with the EBV genome, in particular with the origin of its replication (oriP) (Hussain et al., 2014). The N-terminal region of this factor at a deeper level consists of two DNA binding regions LR1 and LR2 (linking regions) separated by a long unstructured region of glycine-alanine repeats (Jiang et al., 2018). The LRs themselves also have a more subtle structure similar to each other and consist in turn of a glycine- and arginine-rich subdomain (GR) and a region specific to each LR (Jiang et al., 2018). For the C-terminus, such a fine structure is mostly not identified. Between it and the N-terminus, there is also a USP7 binding site and a nuclear localization signal (Hussain et al., 2014).

EBNA1 functions begin with the interaction of its C-terminal domain with the oriP of EBV. The latter occurs within two oriP sites separated by 1 kb or a family of repeats (FR) and a dyad symmetry (DS) element (Reisman et al., 1985). The first contains twenty repeats, each consisting of an EBNA1 binding site (18 bp) and a short AT-rich sequence of 12 bp. The second has only four interaction sites, which are also arranged in pairs and flanked by short telomere-like sequences (Shah et al., 1992). EBNA1 interacts with all of these regions as a homodimer, which is formed by the interaction of each individual monomer with a specific 18-nucleotide site via the C-terminal domain. In addition to the mechanical proximity associated with the location of the DNA-binding sites, the EBNA1 homodimer is additionally stabilized by protein-protein interactions in the region of the same C-terminal domain. Sometimes, in connection with its functionality, this region of EBNA1 is called the DNA-binding and dimerization domain or DBD/DD (Wilson et al., 2018).

Despite the similarity of the mechanism of EBNA1 interaction with FR and DC, the consequences of this interaction are significantly different. In particular, EBNA1 binding to the DS region leads to the recruitment of proteins of the pre-replication complex: origin recognition complex (ORC), cell division cycle 6 protein (CDC6) and replication protein A (RPA) (Norseen et al., 2008; Zhang et al., 1998; Moriyama et al., 2012). These proteins have a certain level of affinity for LR1 and LR2 and subsequently recruit other components of the replication machinery. The previously mentioned telomere-like regions are the reason for the additional recruitment of the TRF2 (telomeric repeat-binding factor 2) into the initiation complex, which also has affinity for ORC (Atanasiu et al., 2006). Thus, the interaction of EBNA1 with DS oriP is necessary for the formation of the pre-replication complex and, accordingly, leads to the initiation of EBV genome replication.

Binding of EBNA1 to the DS region alone is insufficient for precise synchronization of viral episome duplication with cellular genome replication as is typical for EBV (Nanbo et al., 2007). However, the interaction of EBNA1 with the FR region is believed to solve this problem. The mechanism of binding of EBNA1 to FR is generally similar to that in the case of the DS region and also involves the formation of homodimers. The N-terminus of this factor interacts with cellular DNA. The latter occurs due to the presence of the AT-hooks in the GR1 and GR2 regions at the level of the tertiary structure of EBNA1 (Kanda et al., 2013). These structural motifs are a classic way of targeting a specific cellular factor to an adenine- and thymine-rich DNA duplex and are characteristic of many basally expressed cellular transcription factors (Fonfría-Subirós et al., 2012). However, it is not only direct interaction with host DNA that is characteristic of EBNA1. As mentioned earlier, the N-terminal region also serves as a point of organization for many factors of the replicative apparatus, which themselves mostly have some level of affinity for DNA. In addition, nuclear-localized factors such as hEBP2, HMGB2, and RCC1 are directly associated with both host DNA interaction and EBNA1 (Nayyar et al., 2009; Jourdan et al., 2012; Deschamps et al., 2017). Thus, in the case of interaction with the DS region, EBNA1 is able to act as a bridging factor that provides physical binding of the EBV genome to the host chromosome. Along with this, dimers that are localized in both DS and FR are also able to form higher-order structures through the interaction of the LR1 and LR2 domains (Deakyne et al., 2017). This leads to the physical approaching of DS and FR, the final stabilization of the complex and the formation of a loop-like structure of the DNA duplex. Accordingly, after the formation of such a multicomponent nucleoprotein complex, the EBV genome at the point of its replication initiation is actually bound to a specific host chromosome. This allows synchronizing EBV and host DNA duplication in the late S-phase of the cell cycle, as well as evenly dividing the newly synthesized viral genetic material between daughter cells (De Leo et al., 2020).

EBNA1, like all EBNAs, also functions as a transcription factor for both cellular and viral genes. It is now known that this factor, through the recruitment of other cellular DNA-associated proteins, such as CTCF, induces the formation of large loop-like structures in the EBV episome due to the approaching of the oriP FR region, Cp, Qp promoters, and the LMP promoter set (Arvey et al., 2012; Tempera et al., 2011). This is consistent with data on the activity of EBNA1 as a stimulator of transcriptional activity of these regions. (Ceccarelli and Frappier, 2000). And also with the fact that NPM1 (a histone chaperone and transcription activator similar to the pre-replication complex described above) binds to the GR repeats of the N-terminus of EBNA1 (Malik-Soni and Frappier, 2014).

Much less is known about the activity of EBNA1 as a regulator of cellular gene expression, although thousands of sites of interaction of this protein with the host genome have now been identified (Tempera et al., 2016). Among them are IL6R, KDMC4C, EBF1 and MEF2b, which are genes whose products are essential for cell division and differentiation (Tempera et al., 2016). However, the more precise mechanisms of EBNA1′s influence on human genome replication still remain unknown.

Thus, EBNA1 is a major factor required for successful and synchronous replication of viral DNA. In addition, this factor together with other EBNAs is involved in the regulation of transcription of the cellular and viral genomes, although to a much lesser extent compared to more specialized viral proteins.

In summary, the viral genome transcription program (VGTP) at latency IIb ensures complete stabilization of the infection of a susceptible cell. EBNA3 family proteins play a particularly important role in this process. It is these viral proteins that provide the transition from the tight stimulation of proliferation mediated by EBNA2 and, accordingly, MYC to a more complex and large-scale stimulation of proliferation directly regulated by EBV factors. It, in turn, can last for a sufficiently long period without the risk of apoptotic cell death. EBNA1, in this case, ensures the synchronization of viral DNA replication with cellular genome replication and, accordingly, cell division.

## 6 Movement of the infected B-lymphocyte to the regional lymph node. Transcriptional latency program III

Just as EBNA2 activates the transcription of a cluster of all EBNAs with Cp, this early latent factor is also a potent positive regulator of the membrane viral protein family (latent membrane protein or LMP) that includes LMP1, LMP2a and LMP2b. LMP1 and LMP2b proteins are directly regulated by a single bidirectional promoter, and LMP2a by its own separate promoter located at a distance of about 3200 nucleotides upstream in the sequence. At the same time, the LMP1 gene is located on the opposite strand relative to all other latent genes, and the sequences of LMP2a and LMP2b overlap in such a way that their transcripts differ only in the N-terminal region, which contains another additional domain in LMP2a.

As mentioned above, the concentration of LMP proteins, despite the obvious stimulatory effect of EBNA2 immediately after the host cell infection, begins to increase only about 2 days after the onset of infection and gradually reaches a peak only on the 28th day (Baccianti et al., 2023). The actual beginning of the mass production of LMPs is considered to be the transition of the transcriptional activity pattern of the EBV genome from latency program IIb to latency program III.

**Fig. 6:**
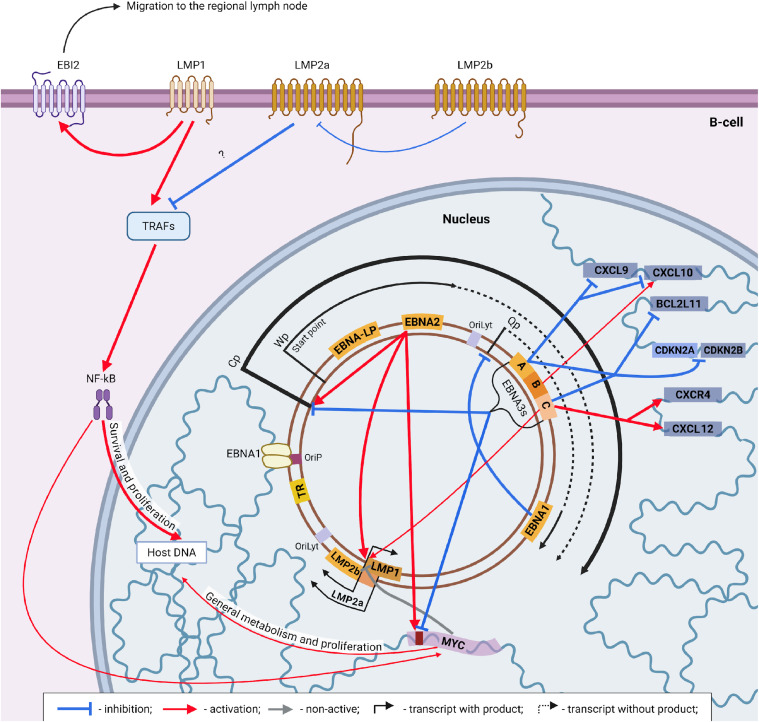
The general scheme of the pattern of mutual influence of EBV and the host cell proteins both at the level of genes and at the level of cellular signaling pathways during the transcriptional latency program III. The thickness of the arrows corresponds to the relative intensity of the influence. Created in BioRender. Zaremba, P. (2025) https://BioRender.com/e0g3z9a.

According to recent data, such kinetics of accumulation of LMPs is explained by the action of the MYC oncogene, which, as mentioned above, reaches its peak concentrations precisely at the beginning of the infection and subsequently decreases due to the influence of EBNA3A and EBNA3C (Fig. 6). The inhibitory effect of MYC is realized by at least two mechanisms. In particular, Mad1 and Max (members of the MYC-mediated transcription regulation cascade) are able to bind to the E-box, a sequence that acts as a site of interaction of host transcription factors with its genome (Sjöblom-Hallén et al., 1999). An E-box-like sequence is located within the promoter upstream of one of the LMP1 transcriptional enhancers. The interaction of Mad1 and Max here results in significant transcriptional silencing of LMP1 and LMP2b, since both these factors have a shared promoter region.

Another mechanism for controlling LMP1 accumulation has been demonstrated. In particular, MYC-induced miRNAs miR17/92 can bind complementary to the 3′ UTR of LMP1 mRNA and, thus, control the rate of degradation of the latter through RNA interference (Forte et al., 2012). At the same time, the degree of affinity of EBNA2 to the bidirectional promoter is identically high both during the period of low LMP1 expression and during the period of its growth and maximization (Price et al., 2018). Similar mechanisms may be present for LMP2a, as the synthesis of the three LMPs is currently thought to be synchronous. Although the more precise features by which the recruitment of transcriptional machinery is finely regulated are still unknown (Chen et al., 2014).

All LMPs are membrane proteins whose main function is the regulation of cellular signaling pathways. In particular, LMP1 consists of six transmembrane helices and a C-terminal cytoplasmic region of 200 amino acid residues. The latter contains two domains CTAR1 and CTAR2 (also called TES1 and TES2) (Soni et al., 2007). With the latest data, a third domain or functional region CTAR3 is also identified between the first two (Šimičić et al., 2024). The transmembrane part of LMP1 provides constitutive trimerization (according to some data, oligomerization) of this factor and, as a result, constitutive activation of the C-terminal cytoplasmic domains. Due to the latter, this factor provides constant activation of a number of signaling pathways, such as NF-κB, MAPK, JNK, JAK/STAT and PI3-K (Kieser and Sterz, 2015). Currently, the most important signaling pathways are those associated with the stimulation of NF-κB, which is one of the key factors in the formation of the immune response and inflammation.

Based on the high similarity of the activation mechanisms of this signaling pathway, LMP1 is considered a direct viral analogue of the CD40 receptor, which is a member of the TNFR family (also called TNFRSF5) and the main coreceptor of B-lymphocytes (Wang et al., 2017). In the case of naive B-cells, activation of this surface factor occurs through trimerization induced by interaction with CD40L. The latter is mostly present on the surface of CD4+ *T*-lymphocytes (Bosmans et al., 2021). The homotrimerization induced in this way leads to the interaction of TNFR-associated factors (TRAFs) with the cytoplasmic domain of CD40, which has two domains for interaction with these proteins: a distal one for TRAF2 and TRAF3/5 (TRAF5 interacts with the receptor mediated through TRAF3) and a proximal one for TRAF6. In the case of LMP1, binding to these TRAFs is provided by the constitutively active domains CTAR1 and CTAR2 (Zhang et al., 2020). TRAFs, in turn, activate different signaling pathways depending on their combination (Bishop et al., 2007). Among them, the most studied and considered to be the most important are the canonical (via TRAF6) and non-canonical (via TRAF2 and TRAF3/5) pathways of NF-κB activation (Rickert et al., 2011). It should be noted that the NF-κB factor is not a single protein, but a family of five structurally and functionally homologous proteins: RelA/p65, RelB, c-Rel, p50, and p52 (Liu et al., 2022), which in the active condition are in a homo- or heterodimeric state.

TRAF6 binding to CD40 (or LMP1) leads to oligomerization of the former in response to trimerization of the latter, which, in turn, causes activation of TRAF6 ubiquitin ligase activity (Yamamoto et al., 2021). Polyubiquitination of TAK1 (TGF-β-activated kinase 1) at K63 leads to the recruitment of TAB2 (TAK1-binding protein 2), which has an affinity specifically for K63-Ub TAK1. Its interaction with TAK1 causes the recruitment of several TAK1s and their subsequent autophosphorylation at Lys158 (Lei et al., 2019). Thus, polyubiquitination in this case serves as a signal for activation rather than for proteasomal degradation, as it usually does. Activated TAK1 phosphorylates IKKβ, which is one of the catalytic subunits of the IKK kinase complex (IκB kinase). This complex also consists of IKKα and NEMO. NEMO is a regulatory subunit that, like TAB2, has an affinity for K63-Ub TAK1, thus performing a regulatory function. Activated IKK phosphorylates IκBα (a constitutive inhibitor of NF-κB) at Ser 32 and 36. Phosphorylation of this factor leads to its recognition by the ubiquitin ligase β-TrCP and rapid polyubiquitination at Lys 21 and 22, which in this case canonically leads to proteasomal degradation of IκBα (Komives, 2023). Thus, NF-κB dimers are released into the cell cytoplasm in a fully functionally active state. Due to the presence of a nuclear localization signal, this factor in its dimeric form quickly enters the nucleus, where it regulates hundreds of genes (Giridharan and Srinivasan, 2018). Among them is the IκBα gene, which is stimulated particularly strongly. Accordingly, the speed of the response implementation and its termination depends on the rate of proteasomal degradation of IκBα and the rate of its synthesis, which is mostly measured in minutes. Thus, initiation of the canonical NF-κB activation signaling cascade causes rapid and potent but short-lived metabolic response (Truhlar et al., 2008).

Instead, the so-called non-canonical pathway of NF-κB stimulation, which is also activated by both CD40 and LMP1, results in a much longer-lasting effect of NF-κB on the transcriptional profile of the cell (Sun, 2012). Binding of TRAF2 and TRAF3 to the cytoplasmic domain of the activated receptor leads to a direct interaction between these two factors. TRAF2 has a certain level of affinity for the ubiquitin ligase cIAP. This results in enhanced ubiquitination of TRAF3 and, consequently, its degradation. It should be noted that in the absence of a stimulus from the activated receptor, TRAF3 is also in a complex with TRAF2-cIAP. In this case, TRAF3 recruits NIK (NF-κB-inducing kinase) to this constitutively active complex, in which NIK is ubiquitinated by the cIAP. This leads to its proteasomal degradation. It seems likely that there is a certain level of constitutive ubiquitination in TRAF3 as well. However, this was not observed in vivo (Yang and Sun, 2015). This is explained by the affinity of the deubiquitinase Otud7b for ubiquitinylated TRAF3. As well as the probable greater activity of cIAP specifically towards NIK, although the exact mechanisms are currently unknown (Yang and Sun, 2015). The consequence of the enhanced proteasomal degradation of TRAF3 is the gradual accumulation of functionally active NIK. The latter, if sufficiently accumulated, begins to more actively phosphorylate residues S866 and S870 of p100. p100, in turn, is a precursor of p52, one of the proteins of the NF-κB group. Its phosphorylation leads to the involvement of the ubiquitin ligase SCF^βTrCP^, which ensures the ubiquitination of K856 (Fong and Sun, 2002). In this case, this post-translational modification leads to partial proteolysis of p100 with the release of p52. After that the latter dimerizes to form fully active NF-κB. It should also be noted that p100, in addition to being an accessible pool of p52, also functions as an IκB-like molecule by binding to NF-κB and sequestering it in the cytoplasm (Derudder et al., 2003). Thus, proteolysis of p100 leads not only to an increase in the amount of NF-κB, but also to its release in a functional form.

NF-κB, as a transcription factor, regulates the cell cycle similarly to MYC. However, MYC exerts its influence through a global change in cell metabolism towards increased catabolic and anabolic processes (including through a large-scale increase in transcription and ribosome synthesis, and in OXPHOS components) (You et al., 2025), as well as direct stimulation of E2F and CDK4, which are regulators of transition between different stages of the cell cycle (Dang, 2013). In contrast, NF-κB more specifically activates cell metabolism (Capece et al., 2022). This factor enhances glycolysis, fatty acid oxidation, and NADPH synthesis. A similar repertoire of activities normally allows activated B-lymphocytes to survive in the dark and light zones of the germinal center during affinity maturation. This is due to the fact that the extremely high rate of their proliferation in the dark zone leads to the formation of a highly hypoxic environment. Accordingly, only cells that produce antibodies with a sufficient level of affinity for the antigen receive a sufficient level of CD40 stimulation from CD4+ *T*-lymphocytes to survive in the light zone of the germinal center (Kennedy and Clark, 2021). For this reason, NF-κB is considered one of the key factors in the formation of the immune response and inflammation (Hayden and Ghosh, 2008). It should also be noted that one of the genes stimulated by this transcription factor is MYC (Kober-Hasslacher et al., 2020). It is assumed that some variable level of stimulation of anabolism and division characteristic of activated B-cells is a consequence of MYC activity (Capece et al., 2022). Along with this, there are studies that postulate the incompatibility of high NF-κB activity with MYC overexpression (Klapproth et al., 2009). This is due to the fact that high levels of MYC, in addition to stimulating DNA damage caused by high proliferation, as well as the synthesis of a number of pro-apoptotic proteins, also suppress the accumulation of the Fas receptor signaling inhibitor protein CFLAR. In contrast, high levels of NF-κB lead to stimulation of Fas receptor synthesis. This, in turn, is manifested in the high sensitivity of the cell to apoptosis induction by, for example, cytotoxic T-lymphocytes, which is especially relevant for activated B-cells in the germinal center where the affinity selection occurs. Moreover, at a certain level, the Fas receptor shows a tendency to self-assembly and, accordingly, self-activation (Mundle and Raza, 2002). Normally, this process is balanced by CFLAR production. Thus, without additional mutations (such as the IGH: BCL-2 translocation characteristic of many lymphomas), simultaneous overexpression of MYC and NF-κB leads to rapid cell self-destruction (McMahon, 2014). Although, as described above, at physiological levels, these signaling pathways are likely to be quite compatible.

Thus, time-separated stimulation of MYC and NF-κB by EBNA2 and LMP1 factors, respectively, allows EBV to avoid the dangerous activation of two mutually exclusive growth-stimulating signaling pathways for non-transformed cells, thereby achieving both cell survival and proliferation at the early stages of EBV infection, as well as its survival within the germinal center.

Another important function of LMP1, although poorly studied compared to that described above, is the stimulation of EBI2 (Epstein-Barr virus induced gene 2) (Daugvilaite et al., 2014). The eponymous product of this gene is a surface receptor for 7α,25-dihydroxycholesterol, i.e. a substance secreted by stromal cells of follicles of secondary lymphoid organs. Interestingly, EBI2 was discovered precisely as a surface protein that is highly expressed in EBV infection (Birkenbach et al., 1993). Only more than 15 years after its discovery its physiological role in the migration of antigen-activated B-lymphocytes to the follicles of regional lymph nodes was identified (Pereira et al., 2009). Thus, the appearance of LMP1 on the cell surface, in addition to affecting the metabolic state of the infected cell and its phenotype, leads to the activation of its movement towards the regional secondary lymphoid organ.

Returning to the intracellular effects of LMP1, it should be noted that the constitutive activity of the CD40 pathways provided by this surface protein, without the expression of other viral factors, leads to the impossibility of germinal center formation in response to immunization (Panagopoulos et al., 2004). This is due to the fact that naive antigen-activated B-lymphocytes during affinity maturation constantly iteratively migrate from the light zone of the germinal center, where based on affinity for the antigen they receive a certain degree of CD40 stimulation from T-lymphocytes, to the dark zone, where they directly divide until the previously stimulated signaling pathways are attenuated and vice versa (Mesin et al., 2016). In the case of constitutive stimulation of proliferation and survival, this process is inhibited accordingly. Additionally, there are studies that show a high activation of the immune response against such LMP1+ *B*-cells (Zhang et al., 2012; Jin et al., 2025).

It is now believed that another EBV surface protein, LMP2A, acts as a regulator of the LMP1-activated signaling cascade during infection. It is not required for EBV-induced B-cell transformation in vitro, which is probably the reason the data available on this protein are limited (Vrazo et al., 2012). But it is highly likely that LMP2A is necessary for the full establishment of latency in vivo.

Like LMP1, LMP2A consists of a membrane and a cytoplasmic part. The first contains 12 transmembrane helices, which ensure the localization of LMP2A. The second, in turn, includes both the C- and N-termini of this protein: the C-terminus has the ability to form intermolecular homotypic interactions, and the N-terminus provides the main functional activity due to the immunoreceptor tyrosine-based activation motif (ITAM). ITAM is normally present in the BCR (B-cell antigen receptor), which is the characteristic and most abundant receptor on the surface of B-cells (Matskova et al., 2001; Yang and Reth, 2016). In naive B-cells, the BCR ITAM is activated by phosphorylation only through antigen binding-induced destruction/recombination of self-inhibited receptor clusters and the consequent recruitment of Lyn kinase to the now accessible ITAM tyrosines. In turn, phosphorylated ITAM is recognized by kinases of the Syk family and leads to the activation of a number of signaling cascades. Among them, the most studied are such global pathways as PLCγ2, PI3K/Akt and MAPK (Wen et al., 2019; Okada et al., 2005). Unlike the BCR ITAM, the tyrosines of the LMP2A ITAM are permanently available for phosphorylation. Accordingly, by analogy, the BCR-stimulated signaling pathways due to LMP2A production should be continuously active in EBV-infected cells approximately one month after the onset of infection. However, along with the clear presence of functional mimicry, recent studies also indicate a slightly different spectrum of changes in the metabolic status of a cell under the action of LMP2A compared with the BCR (Engels et al., 2012). In particular, a recent direct differential study of LMP2A- and BCR-stimulated changes in the phosphoproteome and transcriptome revealed only 12 % overlap of the former, as well as increased expression of 2093 genes upon LMP2A stimulation compared to 247 genes upon BCR stimulation (Fish et al., 2020). Moreover, changes in the phosphorylation pattern, and accordingly in signaling, primarily concerned the degree of phosphorylation of BCR (the measure of phosphorylation of its ITAM decreased). Characteristically, this was accompanied by the simultaneous preservation of the degree of phosphorylation of protein kinases SYK, BTK, and PLCγ2. Transcriptome analysis allowed to identify the ID3 factor as simultaneously stimulated by BCR and LMP2A. This factor is involved in the regulation of the cell cycle during the maturation of B-lymphocytes (Pan et al., 1999). At the same time, LMP2A-specific stimulation of anti-apoptotic genes BCL2L10 and BCL-xL, as well as suppression of pro-apoptotic BIM and BNIP3L were observed.

Another large-scale earlier study also postulates an effect of LMP2A on LMP1-stimulated cell growth through modulation of TRAF (Vrazo et al., 2012). In particular, this study showed that expression of LMP1 in B-lymphocytes, along with impaired germinal center formation, resulted in a twofold increase in TRAF2 (by an unknown mechanism). In contrast, coexpression of LMP1/2A was associated with a complete restoration of physiological TRAF2 levels. However, most striking, according to the researchers, was the restoration of the normal process of germinal center formation disrupted by LMP1 expression upon coexpression of LMP1/2A. Given the absence of apparent impairment of the formation of a specific immune response in the EBV-positive part of the world's population, it is precisely such a clear regulation of LMP1 function by LMP2A that is present during the establishment of EBV infection.

The last member of the LMP family is the LMP2B protein. It is an even less studied EBV factor than LMP2A. In fact, the only reliably characterized aspect of this factor is its structure. As mentioned above, LMP1 and LMP2B are under the control of the same bidirectional promoter. This promoter is located downstream of the LMP2A gene. Thus, transcription initiation from LMP1/LMP2B leads to the production of LMP1 and a truncated form of LMP2A. This form lacks the N-terminus, which is responsible for the protein functionality, but has a full-fledged transmembrane domain and C-terminus. Thus, it is assumed that the probable (but not limited to) function of LMP2B as a derivative of LMP2A is to regulate the function of the latter. This is indirectly confirmed by the localization of LMP2A and LMP2B in the same cellular compartments, as well as the inhibition of LMP2A phosphorylation upon its coexpression with LMP2B (Rovedo and Longnecker, 2007).

In summary, the transcriptional latency program III is the most diverse in terms of the factors involved in the transcriptional program of the viral genome. Based on current data, the additional involvement of surface proteins of the LMP family at this stage allows the virus to imitate the activation of the infected B-lymphocyte and direct it to the regional lymph node. There, by providing permissive stimuli to CD4+ *T*-lymphocytes through a complex pattern of LMP activities, it allows to imitate the full passage of the infected cell through the germinal center. Thus, theoretically, EBV does not require the tissue-specific influence necessary for B-lymphocytes to continue maturation. However, at the same time, there are studies that postulate the presence of infected B-lymphocytes in the follicle with a phenotypic profile completely identical to that of naive B-cells undergoing affinity maturation, namely surface CD10, CD77, CD38, and intracellular AID and BCL-6 (Roughan and Thorley-Lawson, 2009). Accordingly, these infected B-cells, contrary to the previous statement, should undergo a completely normal selection process within the germinal center. Reconciling these two statements, it can be assumed that the establishment of the latency program III is necessary mainly to target the infected lymphocyte to the germinal center while maintaining its high mitotic potential at the expense of most EBNAs and part of LMPs.

## 7 Germinal center theory. Transcriptional latency program IIa

**Fig. 7:**
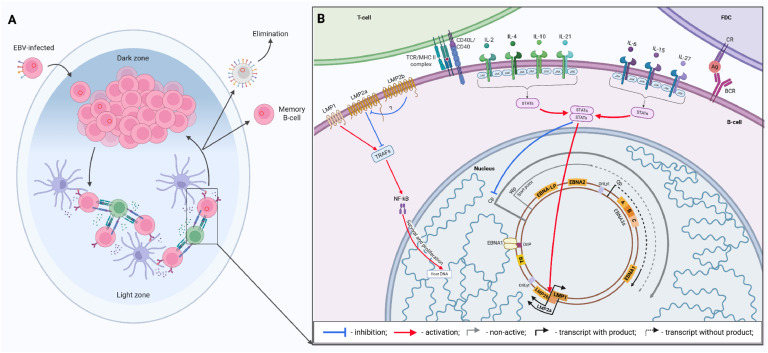
The influence of the germinal center microenvironment on the infected B cell. The process of affinity maturation of activated B-lymphocytes within the germinal center and the location of the EBV-infected B-cell therein (A). The influence of the germinal center microenvironment on the formation of the transcriptional latency program IIa (B). The thickness of the arrows corresponds to the relative intensity of the influence. Created in BioRender. Zaremba, P. (2025) https://BioRender.com/k3j975f.

In the case of intact lymphocytes, the EBV genome expression pattern stabilizes at the level of latency program III, immortalizing the infected cells (Kang and Kieff, 2015). However, available data indicate that in vivo an infected cell changes the EBV genome expression pattern after penetration into the follicle and localization in the germinal center formation zone (Fig. 7, A) (Murata et al., 2021; Price and Luftig, 2015). In particular, all viral factors that depend on Cp activity (EBNA2, EBNA-LP, EBNA3s) are absent in such lymphocytes. This pattern of the EBV genome transcriptional activity has been called latency program IIa or (in earlier studies) latency program II (Young et al., 2007). Currently, the exact reasons and mechanisms for such changes in the transcriptional activity remain unknown. However, based on the limited amount of experimental data, it is still possible to assume certain processes that may occur in the infected cell at this stage and lead to a reduction in the range of viral proteins (Fig. 7, В).

In particular, as mentioned above, the proteins EBNA3a and EBNA3c are largely antagonists to EBNA2. The latter is considered to be the main transcriptional stimulator of Cp and forms a positive feedback loop for its own accumulation. EBNA3s as components of a polycistronic transcript shared with EBNA2 interfere with this process. At the level of latency programs IIb and III, this leads to a fine balance of their total activity for a sufficient level of division stimulation and survival of infected cells. However, when an EBV-infected B-lymphocyte enters the local lymph node and one of its germinal centers, it is exposed to the action of a specific microenvironment, namely cytokines of follicular dendritic cells (FDC) and CD4+ *T*-lymphocytes. One of the most large-scale studies in recent years has somewhat improved the understanding of this process (Liao et al., 2024). Particularly, it was found that most germinal center cytokines affected the transcriptional activity of the virus, although in different ways. All FDC interleukins (IL-6, IL-15 and IL-27) suppressed EBNA2 and, accordingly, EBNA3s without affecting the remaining latent proteins. IL-2 and IL-4 of CD4+ *T*-lymphocytes had a similar effect. In contrast, IL-10 and especially IL-21 (which are also secreted by CD4+ *T*-cells) along with a pronounced suppression of Cp, led to an increase in the expression of LMP1. Interestingly, along with the Cp suppression, IL-15, upon closer examination, stimulated such latent genes as BZLF1 and BMRF1. Attempts to identify the synergistic effect of cytokines that apparently act together in vitro revealed that the combination of IL-4/IL-21 leads to a more pronounced effect of enhancing LMP1 accumulation while maintaining the degree of inhibition of EBNA2 and EBNA3C production at the same level as IL-21.

The effect of interleukins, like most cytokines, is mediated by the JAK/STAT signaling cascade (Delgoffe and Vignali, 2013). In the abovementioned study, scientists suggested that the synergistic effect of cytokines on EBV-infected lymphocytes and the overall effect on the activity of its genome may be mediated precisely by STATs and their combination. Further research confirmed this assumption. In particular, the action of IL-15 led to the accumulation of the phosphorylated form of STAT5, while IL-21 stimulated the phosphorylation of STAT3. At the same time, the use of a JAK inhibitor in the case of IL-15 caused the weakening of its negative effect on Cp activity; and JAK inhibition during IL-21 stimulation led to the weakening of both the inhibition of Cp and the stimulation of LMP1 mediated by this interleukin. Studies on the knockout of the STAT3 and STAT5 genes showed that, along with the significant dependence of the implementation of the IL-15 effect precisely through STAT5, and IL-21 through STAT3, their simultaneous knockout caused a more pronounced disruption of the EBNA2 and EBNA3s inhibition by these interleukins. Although only the disturbance of the STAT3 accumulation was sufficient to block the stimulation of enhanced LMP1 transcription. Taken together, this clearly confirms the role of JAK/STAT in both the inhibition and stimulation of EBV genome transcription. In addition, it is highly likely that homo- and heterodimerization of STAT3 and STAT5 is the process that mediates the synergistic effect of various germinal center cytokines on the transcriptional activity of EBV.

After the transition of the transcriptional program to IIa, LMPs begin to play the main role. This is evidenced, among other things, by the stimulation of LMP1 accumulation by germinal center interleukins, which further aggravates the question of the function of LMPs in the development of the EBV infection. Since infected B-cells demonstrate a morphological profile identical to naive B-lymphocytes of the germinal center, despite the presence of survival and proliferation signals from CD40 and BCR, which are constantly mimicked by LMP1 and LMP2a (Roughan and Thorley-Lawson, 2009). And their immunoglobulin genes show clear signs of class switching and somatic hypermutation (Souza et al., 2005). Together, this indicates the most “natural” and complete passage of affinity maturation by such infected lymphocytes, which in addition accumulate surface proteins, namely functional homologues of those receptors, the activity of which should become a permissive signal for the premature and abnormal termination of this process (meaning LMP1 and LMP2a). The above-described looks like nonsense. However, the situation becomes somewhat better if we rise to a higher level. The number of EBV-infected B-lymphocytes in the germinal center does not exceed 3–4, which is a very small value compared to the typical amount of B-cells that undergo affinity maturation (10^5^ per center) (Roughan and Thorley-Lawson, 2009; Thorley-Lawson, 2015). And also considering the fact that the germinal center is mostly formed by several dozen activated B-cells (Young and Brink, 2021), one of which, obviously, must be infected. Accordingly, the number of lymphocytes, that are EBV-carriers, should be at least several hundred times higher.

The only explanation at the current level of understanding of the molecular biology of EBV, as well as the features of the formation of the germinal center, is a significantly enhanced elimination of virus-infected cells. Taking into account the above-mentioned phenomenon of stimulation of switching between transcriptional programs, mediated by the action of interleukins; and also that normally the secretion of IL-4 and IL-21 is stimulated during the interaction of activated B-cells with T follicular helper cells (Tfh) (Zhang et al., 2012). It seems likely that for full germinal center passage and, consequently, the establishment of EBV latency, the infected cell must fully mimic the antigen-activated B-lymphocyte. This includes the presence of a MHC-II/processed antigen complex on its surface. This antigen may be either a one received and processed by the B-cell before it was infected with EBV, or it may be one of the viral proteins that accumulate during the transcriptional latency program III. The first case seems unlikely because the activated B-cell immediately begins to migrate towards the lymph node, thus moving away from the pharyngeal epithelium. The second seems more likely and can be considered as one of the interesting options for the adaptation of viruses to their hosts. After the formation of the germinal center, the EBV-infected lymphocyte, due to the interaction with Tfh, the action of IL-4 and IL-21, and the support of FDC cytokines, undergoes a change in the latency program from III to IIa. The further selection process probably proceeds according to a completely natural mechanism of iterative affinity maturation, with the only difference being the presence of LMP1 and LMP2a on the B-cell surface. They are intensively accumulated in response to interleukin stimulation received from T-helpers and function in the dark zone. The latter probably leads to an increase in division in such lymphocytes, as well as to their rapid migration beyond the germinal center as memory cells, since LMP1 is capable of inhibiting BCL6 accumulation and stimulating BCL2 accumulation (Panagopoulos et al., 2004; Henderson et al., 1991). The first one is considered a transcription factor stimulated by germinal center interleukins, which ensures the constant position of the B-lymphocyte within the germinal center by inhibiting the accumulation of surface migration molecules (S1PR1, EBI2). And the second one is an anti-apoptotic protein, which, under stimulation, allows B-lymphocytes to survive even in germinal center conditions and transition to a state of resting memory cells (Laidlaw and Cyster, 2021).

Although the direct mechanisms of this process are unknown, the described above explains why there is such a small proportion of infected B-lymphocytes in the germinal center at any given time, but it does not explain how the infected memory cells have a fully completed process of somatic hypermutation. On the other hand, B-cells that have sufficiently active LMPs for the quick exit from the germinal center are likely to be rapidly destroyed by exogenous cytotoxic T-lymphocytes due to excessive presentation of antigens in a functional form on the surface and in the composition of MHC-I (Thorley-Lawson et al., 2013). In addition, the number of activated cytotoxic T-cells is significantly higher in EBV-infected tonsils (Hislop et al., 2005), which also confirms this. Thus, only B-cells that have a sufficiently tight and finely tuned level of LMPs regulation are able to remain within the germinal center for a fairly long time to fully complete the process of somatic hypermutation. As well as they are able to survive throughout the entire period of affinity maturation, while avoiding the risk of becoming plasma cells, determining their own fate only by the pool of memory B-cells.

## 8 Transcriptional latency program I/0. Suppression of reactivation

**Fig. 8:**
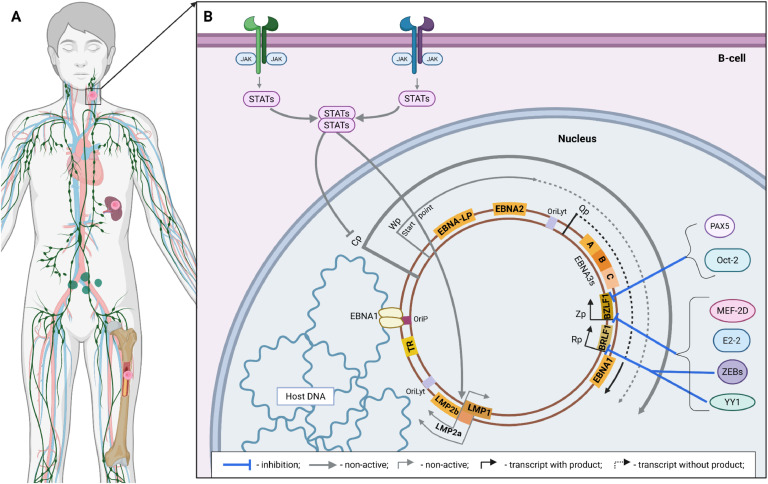
Suppression of the EBV reactivation in infected memory B-cells. Location of infected resting B-cells within the organism (A) and the scheme of suppression of lytic reactivation to maintain the transcriptional latency program I/0 (B). The thickness of the arrows corresponds to the relative intensity of the influence. Created in BioRender. Zaremba, P. (2025) https://BioRender.com/u4ydxvl.

After leaving the germinal center, the EBV-infected memory B-cell is eventually deposited in the spleen, red bone marrow, or peripheral tissues (Fig. 8, A) (Palm and Henry, 2019). Some also remain circulating with the bloodstream. There is currently insufficient direct experimental data on the molecular processes that occur at this stage. However, it is known that infected memory B-cells either express only EBNA1 from the entire range of viral proteins, or none at all. These two variants of the viral genome expression pattern are called latency program I and latency program 0, respectively (Fig. 8, B) (T. Murata, 2023). The suppression of LMPs at this stage is likely due to the lack of activating effects of germinal center cytokines as described earlier. Thus, EBV-infected memory B-cells, while in their depots, do not express highly immunogenic surface proteins of viral origin. This allows such B-lymphocytes to avoid the likely long-term elimination due to the action of cytotoxic T-cells. Moreover, even EBNA1, as a factor vital for the synchronization of viral genome replication, is present in the memory cell only in the case of a rare division (Lyu et al., 2025). As a result, after completing all the complex and coordinated with the host's resistance mechanisms stages of establishing latency, EBV acquires the ability to persist in the pool of memory B-cells, remaining almost completely invisible to the immune system as long as such a cell is alive (i.e., for an almost unlimited period) (Latour, 2025).

During this period, cellular transcription factors YY1, E2–2, MEF-2D, ZEBs and others suppress the immediate early lytic genes BZLF1 and BRLF1 (Kenney and Mertz, 2014; Biswas et al., 2025). These genes are located in the intronic region of EBNA1 and have the opposite orientation to it (Murata et al., 2021). Their products are the transcription factors Zta and Rta, which are transactivators of lytic protein promoters. Сellular factors YY1 and E2–2 are able to bind to cis-regulatory regions (ZIV and HI, respectively) upstream of Zp (Zta protein), thus suppressing the activity of this promoter (Thomas et al., 2003). Normally, these proteins are widespread transcription factors involved in B-cell function (Kleiman et al., 2016; Zhou and Murre, 2021). The transcription factors ZEB1 and ZEB2, on the other hand, are capable of binding to the ZV and ZV′ regions that directly surround the BZLF1 transcription initiation site (Yu et al., 2012). ZEBs are normally important factors in the differentiation of B-lymphocytes (Scott and Omilusik, 2019). MEF-2D binds to three upstream sites (ZIA, ZIB and ZID) thereby recruiting class II HDAC complexes (Gruffat et al., 2002). The latter leads to deacetylation of histones and silencing of the BZLF1 translation start site. This transcription factor, like the others, is involved in differentiation processes, including in B-lymphocytes (Szeto et al., 2024). YY1 and ZEB1/2 also inhibit the activity of Rp (Rta protein) by binding to cis-suppressor regions upstream, although data in this direction are somewhat limited (Kenney and Mertz, 2014). Thus, transcription factors widely present in differentiated B-cells act as inhibitors of EBV lytic reactivation.

In addition to transcriptional inhibition of EBV reactivation, this process is also regulated at the protein product level. In particular, the transcription factors PAX5 and Oct-2, which are actively involved in all stages of B-lymphocyte development except the plasma B-cell, interact with Zta, which leads to a decrease in its transcriptional activity (Robinson et al., 2012; Raver et al., 2013). The involvement of such processes in lytic reactivation is also confirmed by the fact that the mutant EBV with a constantly active Zta does not always immediately enter the lytic cycle (Kenney and Mertz, 2014).

## 9 Reactivation of EBV. The immediate early stage of the lytic cycle

Despite the clear suppressive effect of proteins of resting memory B-cells, some of these EBV cellular reservoirs are constantly undergoing lytic reactivation of the pathogen. This is associated with the obvious need of the virus for spread and is normally balanced by the elimination of such cells by CD8+ lymphocytes. The latter is confirmed by the presence of such a widespread disease as EBV-associated post-transplant lymphoproliferative disorder (PTLD), which is a consequence of large-scale uncontrolled proliferation of infected B-lymphocytes (Gupta et al., 2020). A special risk group is considered to be EBV-seronegative recipients, who in the post-transplantation period will almost certainly have lymphoproliferative disorders both in the case of EBV damage and in the case of organ transplantation from an EBV-seropositive donor (Sampaio et al., 2012). The main cause of PTLD is the severe immunosuppression that always accompanies organ transplantation, thus disrupting the delicate balance between the pathogen and the host's defense systems.

**Fig. 9:**
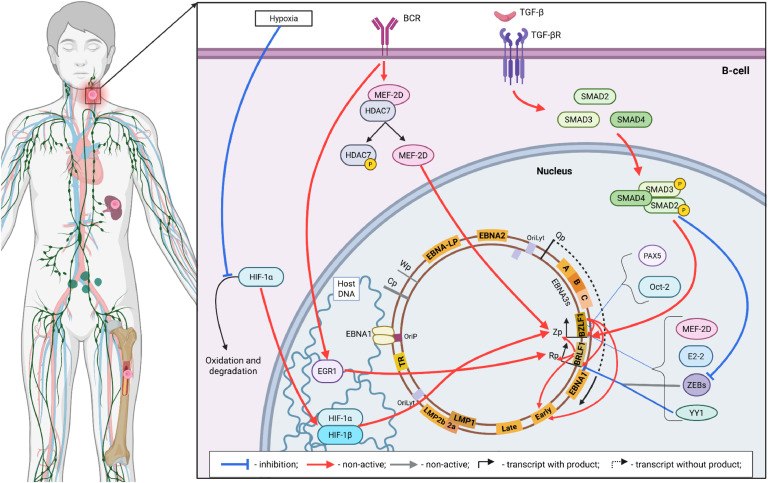
The general scheme of EBV reactivation during stimulation of an infected memory B-cell through BCR, TGF-βR and hypoxia-mediated signaling pathways. The thickness of the arrows corresponds to the relative intensity of the influence. Created in BioRender. Zaremba, P. (2025) https://BioRender.com/k16q0ub.

There are currently no precise, large-scale studies that reveal the direct mechanisms of physiological reactivation of EBV. However, there are studies that demonstrate a significant contribution to this process of BCR cell signaling (Fig. 9). As it was mentioned before, BCR activation leads to rapid and powerful activation of signaling pathways, namely JNK, MAPK/p38, ERK, PKC, PKD, and PI3K/AKT (Kenney and Mertz, 2014). All of them are generally tightly interwoven kinase signaling cascades. MEF-2D, which has been mentioned as a transcriptional inhibitor of BZLF1, is one of the targets of these signaling pathways (Bryant and Farrell, 2002). The dephosphorylated form of MEF-2D has a higher affinity for DNA and the ability to recruit transcription initiation factors. In its base form, this factor is associated with transcription corepressors such as HDAC7. The phosphorylated form of the latter leaves the nucleus, which leads to the release of MEF-2D as a transcription activator (Li et al., 2001; Yasuda et al., 2008). Another consequence of activation of the BCR and the corresponding kinase pathways is the accumulation of EGR1, which is one of the positive regulators of transcription and in tandem controls the final differentiation of the B-cell into a plasma cell (Gururajan et al., 2008). This factor has two binding sites within Rp. Thus, stimulation of the BCR by the appropriate antigen results in simultaneous upregulation of both Zp and Rp.

Other studies show that TGF-β and its canonical signaling cascade are also involved in the reactivation of EBV (Fig. 9) (Iempridee et al., 2011). The main mediators of signal transduction in this case are Smad proteins, which in their unphosphorylated form are localized in the cytoplasm. However, after phosphorylation caused by activation of the TGF-β-dependent receptor kinase complex, regulatory members of this family (in particular SMAD2 and SMAD3) form a heterotrimeric effector complex with SMAD4, which translocates to the nucleus (Deng et al., 2024). Further binding to the promoter region at no less than five sites leads to activation of BZLF1 transcription. It is interesting that SMAD4 competes for binding to ZEBs at one of the sites, which, in addition to the activator function of SMAD4 itself, at a certain level of its accumulation leads to additional overstimulation of Zp.

Hypoxia also highly likely directly leads to the reactivation of EBV (Fig. 9). This is due to the termination of routine degradation of HIF-1α (one of the hypoxia-induced factors) (Jiang et al., 2006). This transcriptional regulator is normally degraded by proline hydroxylation and subsequent polyubiquitinylation under sufficient oxygen. However, under reduced oxygen concentrations, this process is correspondingly slowed down, and HIF-1α accumulates. This allows it to transition to an active form by forming a complex with HIF-1β and stimulating the accumulation of Zta by binding to the promoter region.

Thus, being in a latent state in various pools of resting memory B-cells, EBV (or rather its lytic reproduction) is suppressed due to the involvement of ubiquitous transcription inhibitors characteristic of cells at this stage. In the case of changes in the chemical environment of such infected lymphocytes lytic reactivation of EBV occurs. The mentioned changes can be, for example, due to the B-cell interaction with an antigen, or the influence of TGF-β synthesized by cells in the area of ​​acute inflammation, which is often accompanied by a local decrease in oxygen concentration. (Deng et al., 2024; Taylor and Colgan, 2017). The described phenomenon is further confirmed by the ability of the virus to reactivate in vitro in response to the action of 12-O-tetradecanoylphorbol-13-acetate (TPA), sodium butyrate, anti-Ig, TGF-β, as well as hypoxia directly (Hongde et al., 2016).

Zta and Rta, at a certain concentration, begin to actively stimulate the transcription of early genes of the lytic cycle, cross-transcription of each other, as well as directly themselves. In particular, Zta is able to recognize Zp and Rp at sites ZIIIA and ZIIIB, as well as ZRE1, ZRE2 and ZRE3, respectively (Hongde et al., 2016). Rta, on the other hand, although it is capable of enhancing both its own transcription and that of Zp, acts more indirectly (Yang and Chang, 2013). To stimulate both its own transcription and Zta transcription, this transcription factor involves a number of cellular factors, among which MCAF1 can currently be considered the main one (Lin et al., 2014). Data on this protein and its normal function are currently limited. However, it is known that this transcription factor is associated with cell division and is also able to interact with proteins such as Sp1 and RNA polymerase II (Sasai et al., 2013). Sp1, in turn, is a classical and context-dependent transcription factor (Zhou et al., 2024). The binding sites for this factor are present in both Zp and Rp. Thus, Rta in the case of self-stimulation or stimulation of Zp acts as a factor in the assembly of a coactivator complex based on Sp1. Although there is also an opinion that the influence of the mechanisms described above and several others found in the literature is not the main way of activating Zp and Rp Rta. In particular, based on the fact that there is a large number of unexplored RRE (Rta response elements) in the composition of the EBV episome, it is postulated that Zp and Rp can be positively regulated by a kind of trans-elements thousands of nucleotide pairs from these promoter sites (Heilmann et al., 2012).

Summarizing this stage, it is quite obvious that after a sufficiently regulated and demanding process of EBV reactivation, further events become practically irreversible and continue until the death of the host cell.

## 10 The early stage of the lytic cycle. EBV genome replication

Zta and Rta, after sufficient accumulation, begin to stimulate the promoters of the so-called early EBV genes more and more actively in tandem. It is currently believed that the most important of them are BALF5, BMRF1, BALF2, BBLF4, BSLF1, BBLF2/3, BKRF3, BGLF4, BARF1, BALF1, BHRF1, BDLF3.5, BDLF4, BVLF1, BGLF3, BFRF2, and BcRF1 (Su et al., 2014; Hoebe et al., 2018; Sun et al., 2015). Although this is not actually a complete list (Murata, 2018). Among the studied ones, the gene products BALF5, BMRF1, BALF2, BBLF4, BSLF1, and BBLF2/3 are components of the virus-encoded lytic replicative machinery of EBV. BALF5 encodes a DNA polymerase, BMRF1 is a dsDNA-binding protein and a factor enhancing BALF5 processivity, BALF2 ​​is an ssDNA-binding protein, BBLF4 is a helicase, BSLF1 is a primase, and BBLF2/3 is a linker protein of the helicase-primase complex. The BKRF3 gene instead encodes an uracil-specific DNA glycosylase, BGLF4 is a protein kinase, BARF1 is a viroreceptor for M-CSF, and the products of BALF0, BALF1 (also known as BALF0/1) and BHRF1 are the BCL2-like antiapoptotic factors. BDLF3.5, BDLF4, BVLF1, BGLF3, BFRF2, and BcRF1 are proteins of the viral preinitiation complex (vPIC) required for the synthesis of late EBV factors (Aubry et al., 2014).

The main process initiated at this substage of the EBV life cycle is the replication of its genome. During the lytic cycle, this process begins at an origin of replication different from the latent one. More precisely, at two identical origins located at a distance of about 100 kb, i.e., at approximately opposite sides of the viral episome (Hammerschmidt and Sugden, 2013). According to the fact that oriP is located near one of the TRs and is depicted on the linearized diagrams of the EBV genome on the left, these origins are called oriLyt_L_ and oriLyt_R_ (Yetming et al., 2020). Some of the genes mentioned above were named based on their location relative to lytic origins of replication. Each oriLyt consists of two key regions located within the origin at a distance of ∼0.5 kb. In total, an oriLyt is about 1 kb long. OriLyt_L_ is flanked by two oppositely directed genes BHLF1 and BHRF1. A key element upstream of OriLyt_L_ is located in the BHLF1 promoter region and is characterized by the presence of four Zta binding sites. A similar element is also found in oriLyt_R_ within the promoter region of the BHLF1 paralog gene LF3 (Yetming et al., 2020). Although the BHRF1 gene promoter is not technically a part of oriLyt_L_, it contains a single Zta binding site. There is evidence, albeit outdated, that this region of the genome is also capable of significantly accelerating the replication process (Hammerschmidt and Sugden, 1988). The mentioned key downstream element is closer to the central part of oriLyt and is surprisingly conserved. It consists of about 40 bp and is the binding site for Sp1, Sp3, and ZBP-89 (Baumann et al., 1999).

According to current ideas, at the first stage of EBV genome replication, the Zta protein interacts with its corresponding sites within the oriLyt (Sugimoto, 2022). This factor then recruits the BBLF4 helicase, which in turn is a part of the BBLF4/BSLF1/BBLF2/3 helicase-primase complex (Liao et al., 2001), thus, involving BBLF2/3 as a linker protein and BSLF1 as a primase. Stabilization of the complex in this case is also provided by direct interactions between Zta and BSLF1/BBLF2/3. At this stage, induced DNA melting and recruitment of BALF2 ​​to protect the ssDNA from endonucleases probably occur. BMRF1 also has an affinity for Zta, joining the complex and recruiting BALF5 (Baumann et al., 1999). Thus, Zta can theoretically be considered as the main organizer of the virus-specific replicative machinery of EBV. This is supported by the affinity of Sp1 and ZBP-89 for BMRF1/BALF5 (Liao et al., 2005). Accordingly, the full replicative complex (often called the replisome) will assemble only at the oriLyt region.

**Fig. 10:**
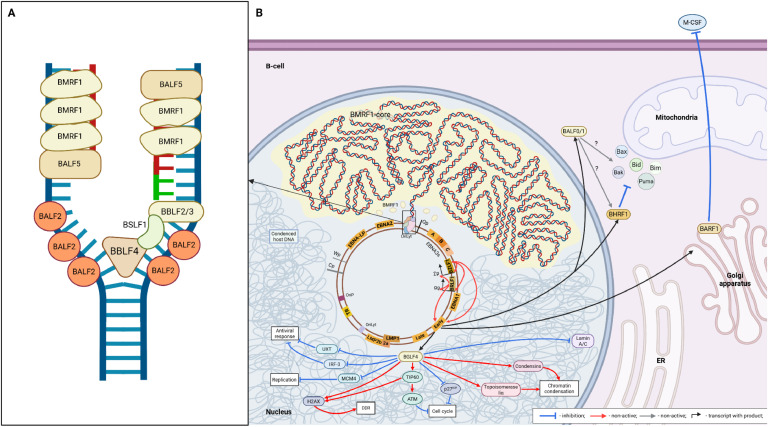
The patterns of the influence of early EBV lytic factors on cellular organization, proliferation, and apoptosis. (A) The structure of the EBV replicative machinery. (B) The overview of the infected B-cell at an early stage of the EBV lytic cycle. The thickness of the arrows corresponds to the relative intensity of the influence. Created in BioRender. Zaremba, P. (2025) https://BioRender.com/j3oojlb.

In general, the replication process is considered to be conservative among all herpesviruses and is one of their taxonomic features (Fig. 10, А) (Weller and Coen, 2012). A classical replication fork is formed as a result of the replisome assembly with simultaneous partial melting of the duplex and priming of the leading and lagging strands. Thus, both strands of viral DNA are produced simultaneously. Their association forms a newly created double-stranded, but linear form of the EBV genome. The movement of the replicative machinery by the episome is considered to be cyclic, which ensures the production of concatemeric DNA. It is mostly an intermediate form of the existence of the viral genome, which is essentially a long, in this case, double-stranded nucleic acid molecule. The latter contains repeating fragments corresponding to the synthesis template.

In addition to the proteins of the replicative machinery itself, the replisome also contains the BKRF3 factor (Su et al., 2014). Due to its ability to catalyze the hydrolysis of the glycosidic bond, this enzyme, like uracil-DNA glycosylase and similar to its cellular counterparts, is involved in the repair of viral DNA. This is considered an important process, given the size of the EBV genome, as well as the massiveness of lytic viral replication (Lu et al., 2007). In addition, the conversion of cytidine to uracil by hydrolytic deamination is a constant process due to the relatively low stability of this pyrimidine (Tremblay and Wagner, 2008). Thus, the accumulation of deoxyuridine nucleotides in the composition of the long-term existing EBV episome is a highly probable process.

There are also studies that indicate a direct effect of BKRF3 on replisome processivity (Su et al., 2014). In particular, Su at all. showed that BKRF3 is able to bind to BMRF1 and BALF5. In the first case, the complex is probably formed before the translocation of the glycosylase into the nucleus, since BKRF3 does not have its own nuclear localization signal. In addition, the two-fold slowdown in transcription caused by the absence of BKRF3 can be eliminated by the presence of a mutant enzymatically inactive form of this protein. Thus, it is assumed that in addition to the reparative function, BKRF3 can also perform a complex-stabilizing function in the replisome.

BGLF4 is another product of early lytic transcription of EBV. An interesting feature of BGLF4 as an early product is its presence in the EBV tegument. In general, based on what described earlier, kinase cascades occupy a special place in the EBV life cycle. BGLF4 recognizes Ser/Thr-Proline motifs, which makes it a homolog of cellular cyclin-dependent kinases (CDKs) (Kawaguchi et al., 2003). Thus, this factor is able to phosphorylate numerous cellular and viral substrates (Massacci et al., 2023; Lee and Chen, 2021). In particular, BGLF4 phosphorylates such cellular factors as UXT, IRF3, TIP60, p27KIP, H2AX, condensins, Topo II, MCM4, and lamin A/C (Fig. 10, B).

UXT is a common factor among different cell types. This factor, in addition to other things, ensures the implementation of the function of NF-κB (p65) by prolonging its functioning in the cell nucleus after activation and translocation (Sun et al., 2007). Phosphorylation of UXT leads to a weakening of the NF-κB/UXT interaction. This inhibits the synthesis of all NF-κB-dependent proteins (Chang et al., 2012). Including factors of the innate antiviral response (Db and Jl, 2006; Taylor and Bresnahan, 2006). This is obviously important in the level of the lytic cycle of EBV or in other words, during the period of mass production of both the viral genome and proteins.

Phosphorylation of IRF3 completes the picture of counteracting the immune response. Actually, IRF3 is one of the main transcription factors responsible for activating the transcription of both IFN-β and other IFN-stimulated genes during viral infection (Morin et al., 2002). Activation of this factor normally involves its phosphorylation at S385, S386, and the serine‑threonine cluster 396–405. However, BGLF4 phosphorylates it at S123, S173, and T180, which are amino acids that form part of the linker region between the DNA-binding domain and the dimerization domain of IRF3 (Wang et al., 2009). This leads to a decrease in the activity of this protein as a transcription factor. Interestingly, the formation of a functional initiation complex for IFN-β transcription activation requires the involvement of NF-κB (Hiscott, 2007).

TIP60 is another factor phosphorylated by BGLF4 during the lytic phase of the EBV life cycle (Li et al., 2011). In the cell, this lysine acetyltransferase is able to acetylate ATM, H2AX, p53, histones H4 and H2, Aurora B1, MRN, and NuA4 (Zohourian et al., 2024). That is, a fairly wide range of substrates, among which the most important in the context of viral reproduction is considered to be the acetylation of the ATM kinase. Autophosphorylation of this factor due to acetylation is a sufficient event to trigger DDR by phosphorylation of BRCA1, p53, chk2, and chk1 (Moslemi et al., 2021). Phosphorylation of chk2, which is specific to ATM, leads to phosphorylation and subsequent degradation of the phosphatases Cdc25A and Cdc25C (depending on the life cycle stage) by the proteasome. This, in turn, leads to the so-called arrest of the cell cycle in S-phase (Zannini et al., 2014). The above-mentioned phosphorylation of BGLF4 p27KIP completes the picture of inhibition of cell division due to the ubiquitinylation of p27KIP (a CDK2 inhibitor) caused by this process (Iwahori et al., 2009).

Inhibition of cell division by EBV is accompanied by prophase-like condensation of cellular genetic material (Lee et al., 2007). This process is also mediated by BGLF4 through phosphorylation of condensins and topoisomerase IIα. These factors ensure the condensation of chromatin into chromosomes during mitosis. The former, when phosphorylated, increase their affinity for DNA and provide additional supercoil turns and probably serve as the basis for the chromatid in the future (Hirano, 2012). Topoisomerase Iiα is required to get rid of the possible knot structures that appear during chromatin condensation (Dyson et al., 2021). It initiates dsDNA breaks at sites of tension, followed by the passage of one strand through the break site of the other.

Simultaneously, BGLF4 phosphorylates and TIP60 acetylates histone H2AX. This subtype of H2A is found in 5–25 % of histone octamers (Giglia-Mari et al., 2011). Its phosphorylation by ATM during cellular life is also associated with the initiation of DDR. The phosphorylated form γH2AX is considered to be a key factor that recruits components such as MRE11/NBS1/RAD50, MDC1, 53BP1, and BRCA1 to the site of DNA damage (Podhorecka et al., 2010). Acetylation is important for limiting the scale of response to genome damage, and is also likely necessary for the full recruitment of the MRE11/NBS1/RAD50 sensor complex to the DDR site (Ikura et al., 2015). However, given that, in addition to direct phosphorylation of BGLF4, TIP60 also indirectly stimulates the accumulation of γH2AX, the stage of recruitment of DDR effector factors seems to be a privileged point of EBV influence in this context. In general, the above-described is logical, since the consequence of the massive pile up of the viral genome is the accumulation of a large number of DNA damages. This is especially true for naked double-stranded ends, which are highly reactive for human cells, but a characteristic phenomenon for viral reproduction (Hau and Tsao, 2017).

The substrate of BGLF4 is also MCM4 (a component of the MCM complex), which is the main helicase of the replicative machinery during DNA replication in S-phase (Kudoh et al., 2006; Bell and Botchan, 2013). Phosphorylation of MCM4 at the CDK site, unlike phosphorylation of Cdc7, leads to inhibition of the formation of the functional MCM4/6/7 complex, thus preventing the initiation of host genome replication in S-phase (You and Masai, 2024).

Phosphorylation of lamin A/C by BGLF4 completes the picture of the effects of this viral kinase on the host cell (Lee et al., 2008). Lamins A/C, together with lamins B1 and B2, form the submembrane nuclear cytoskeleton, thereby maintaining shape and volume of the cell nucleus (Wesley et al., 2024). Phosphorylation of lamins A/C by CDK at the beginning of mitosis and by protein kinase C (PKC) due to, for example, activation of pro-apoptotic signaling pathways during interphase leads to depolymerization of these lamins (Torvaldson et al., 2015). And this consequently leads to a decrease in the rigidity of the nuclear membrane. It should also be noted that BGLF4 has less selectivity, in this case, which leads to a more powerful phosphorylation of lamins compared to the physiological level (Lee et al., 2008).

Thus, only one early factor of EBV, kinase BGLF4, leads to a number of profound metabolic and structural consequences for the host cell. Functional mimicry of this protein with CDK modified for constant influence allows the EBV to stop the cell life cycle at the level of S-phase as the richest in resources available for synthetic processes. It also allows to condense nuclear chromatin, releasing additional volume for viral replication and slowing down transcriptional processes, and also to disable the replication of the cellular genome characteristic of the S-phase. Thus, EBV removes the only competitor for the resources necessary for the mass accumulation of viral proteins and DNA, which is the host cell. Phosphorylation of lamins with their subsequent depolymerization at this stage is a preparation for the successful formation of virions at later stages of EBV life cycle and their transport to the cell membrane.

However, BGLF4 phosphorylates not only cellular proteins, but also EBV factors such as BMRF1, EBNA2, EBNA-LP, BZLF1, as well as BGLF4 itself (Gershburg et al., 2007). Phosphorylation of EBNA2 and EBNA-LP, given their expression at the early stages of establishing the latent type of EBV transcriptional activity, is likely to occur by BGLF4 present in the virion tegument and leads to some initial decrease in the activity of these factors (Yue et al., 2005). The role of autophosphorylation is currently poorly understood, although it is possible that this posttranslational modification is necessary for the detection of increased activity of cellular phosphatases. In contrast, the consequences of phosphorylation of Zta by BGLF4 are known better. Such modification of Zta leads to the formation of a heterodimeric Zta/BGLF4 complex with reduced activity, at least for the activation of transcription with BZLF1 (Asai et al., 2009). It is likely that phosphorylation has a similar effect on the activity of Zta as an activator of transcription of early EBV genes, which explains their extreme accumulation. BGLF4 also phosphorylates BMRF1, which is one of the factors of the EBV replicative machinery. Although there is no objective data on the effect of this modification on the activity of BMRF1 as a factor of the processivity of the replicative machinery, it is likely that the phosphorylated form provided by this kinase is ubiquitous and functionally active (Gershburg and Pagano, 2002). There are also data that significantly expand the range of BGLF4 substrates, especially regarding proteins of the replicative machinery (Zhu et al., 2009). However, the information currently available is insufficient for any possible further assumptions and statements.

Thus, BGLF4, in addition to its crucial contribution to the control of cellular metabolism, also plays a role in regulating the functioning of EBV lytic factors, especially in limiting the accumulation of early lytic phase proteins. Although it should be noted that the available data do not fully clarify this issue.

The last early lytic factors considered here are the proteins BARF1, BALF0/1 and BHRF1. The former is a membrane-associated peptide that is glycosylated and loses its hydrophobic N-terminus during maturation (Lo et al., 2020). This leads to its excretion into the intercellular space in a functionally active hexameric form, where it binds to M-CSF. The latter is a macrophage colony-stimulating factor that is secreted at a basal level by the vascular endothelium and is necessary for the differentiation of mononuclear immune cells, as well as for their successful performance of phagocytic and immunomodulatory functions in the area of ​​inflammation (Dai et al., 2004; Hoebe et al., 2012). BARF1 interaction with M-CSF leads to functional inactivation of the latter (Elegheert et al., 2012). In addition, this viral protein, through a currently unexplored mechanism, is capable of enhancing the proliferation of various cell types (Sall et al., 2004). It is known that these mechanisms must involve surface receptors that will activate downstream signaling pathways that ultimately lead to a significant increase in the transcription of anti-apoptotic Bcl-2, cyclin D1, miR-146a, c-Myc and/or NF-κB (Lo et al., 2020).

BALF0/1 and BHRF1 factors are direct homologs of Bcl-2 family proteins (Fu et al., 2013). BHRF1 is a 21.9 kDa protein that contains three Bcl-2 family domains: BH1, BH2, and BH3. This viral protein, similar to cellular anti-apoptotic proteins, is able to sequester both the pro-apoptotic BH3-containing regulatory proteins Bim, Bid, and Puma, as well as the effector proteins Bak and Bax (Foight and Keating, 2015). This leads to the inhibition of apoptosis at two complementary levels: at the level of the release of effectors Bak and Bax from the influence of anti-apoptotic members of BCL-2; and at the level of the assembly of the pore complex necessary for the initiation of MOMP. The latter is important if the concentration of Bak and Bax is sufficient to overcome the first barrier (Caria et al., 2012). Overall, BHRF1 is a well-studied factor that is being considered as a potential target for the development of antiviral drugs.

Much more controversial are the data on BALF0/1 in the context of counteracting apoptosis of the infected cell. Only recently, based on the appearance of specific IgG in patients, the exact presence of BALF0/1 has been confirmed (Z. Shao et al., 2019). Theoretically, based on the presence of two start codons within the BALF ORF, there should be two transcripts, a longer one with an additional fragment at the N-terminus (BALF0) and a shorter one (BALF1) (Bellows et al., 2002). However, most studies have focused on BALF1. This is likely due to the presence of the Kozak sequence right next to the second methionine codon, making it a more likely target for subsequent translation initiation (Z. Shao et al., 2019). In the context of this protein, it was determined that serum-dependent cells transfected with a BALF1 vector system require much lower serum content in the medium compared to controls, although no effect on the division rate was observed (Cabras et al., 2005). It was also shown that transfection of cells with both BAX- and BALF1-producing vectors resulted in the death of transfectants, whereas transfection of BAX and BHRF1 did not (Bellows et al., 2002). Thus, the probable anti-apoptotic function of BALF1 is clearly weaker compared to BHRF1. On the other hand, recent studies on the effect of BALF0/1 on autophagy showed a stimulatory effect of each of these transcripts on the number of autophagosomes in the cell, which in the case of BALF1 was also manifested under the action of chloroquine. Simultaneous exposure to BALF0, BALF1 and chloroquine did not lead to stimulation of autophagy (Z. Shao et al., 2019). Accordingly, specific interactions are likely even in the cognate pair BALF0 and BALF1. Overall, based on the limited data, it can only be clearly stated that BALF0/1 plays a role in modulating apoptosis during EBV infection. This role is likely to be largely regulatory for both cellular BCL-2 and BHRF1, although it is clearly important, given the significant conservativeness of BALF0/1 (Z. Shao et al., 2019).

Thus, already at the beginning of viral reproduction, intracellular pro-apoptotic stimuli caused by powerful replicative processes, morphological and biochemical rearrangements, are significantly inhibited by BALF0/1 and BHRF1. BARF1, at the same time, allows to weaken the immune response by reducing the activity of monocytes in the area around the infected B-cell.

## 11 The late stage of EBV lytic cycle. Virion assembly and release

As described earlier, after the accumulation of a tolerable level of replicative machinery proteins (i.e., fairly quickly after reactivation), massive synthesis of viral concatemeric dsDNA begins, where each currently available EBV episome serves as a template. DNA accumulation is accompanied by the assemblage of replicative machinery proteins, as well as auxiliary factors directly in the area of ​​​​the original template. Thus, a replication compartment (RC) is formed, which is a clearly separated zone of massive viral replication, transcription of lytic genes and virion assembly (Yetming et al., 2020). In the case of EBV, RC can occupy up to 30–35 % of the nuclear volume in the later stages of infection (Nagaraju et al., 2019).

A special role in the formation of RC is played by BMRF1. Due to its affinity for dsDNA, this protein performs not only the function of the BALF5 processing factor, but also binds the majority of viral DNA (Sugimoto et al., 2019). At the same time, BMRF1 is able to make homotypic interactions, which probably works in favor of the formation of a dense nucleoprotein conglomerate within the RC, additionally protected from the action of, for example, cellular endonucleases (Murayama et al., 2009; Sugimoto et al., 2011). This compact conglomerate is often called the BMRF1 core.

**Fig. 11:**
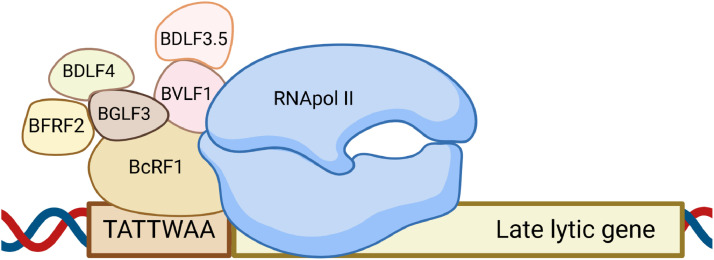
The schematic structure of late lytic genes and the virus-induced transcription complex present at the late lytic stage of the EBV cycle. Created in BioRender. Zaremba, P. (2025) https://BioRender.com/05uz96w.

The promoter region of the late EBV genes is significantly different from that of the early genes (Fig. 11). Transcription of these ORFs does not depend on the 5′-enhancer elements required for transcription initiation of all other ORFs. Instead, it contains a non-canonical TATT box, or more precisely, the sequence TATTWAA (Dremel and Didychuk, 2023), which is recognized by the viral protein BcRF1 (Gruffat et al., 2012). This viral TATA-box binding protein (TBP) is capable of direct interactions with human RNA polymerase II, compared to cellular TBP (Aubry et al., 2014). Based on data on ORF24 (a homolog of BcRF1 in Kaposi's sarcoma-associated herpesvirus (KSHV)), this process is likely to occur through interaction with RPB1, the largest subunit of Pol II (Davis et al., 2015). BcRF1 is bound by BGLF3, which in turn has affinity for BVLF1, BDLF4, and BFRF2 (Dremel and Didychuk, 2023; McCollum et al., 2023). Thus, BGLF3 functions as a structural protein for the formation of the viral preinitiation complex (vPIC). The only vPIC factor that does not directly bind to BGLF3 is BDLF3.5. This protein is recruited to the initiation complex through interaction with BVLF1 (Castañeda and Glaunsinger, 2019). Based on the data on Pol II accumulation on human cytomegalovirus DNA and a parallel slowing of elongation in the absence of UL79, it seems likely that the role of BVLF1 is not limited to transcription initiation (Dremel and Didychuk, 2023). At the same time, given that BDLF3.5 interacts with the rest of the vPIC components exclusively through BVLF1 (BVLF1 binds to BDLF4 and BFRF2, in addition to BGLF3 and BDLF3.5), and is similarly required for transcription, its role as a regulator of BVLF1 in performing the initiation and/or elongation function seems likely (Didychuk et al., 2020). BDLF4 and BFRF2 are also required for vPIC assembly and function. Essentially the only information available about them, mostly based on homology, is their apparent requirement for transcription initiation, as well as their demonstrated interaction with each other and the other factors discussed here (Aubry et al., 2014; Brulois et al., 2015). In summary, it is worth emphasizing that the mechanism of transcription initiation of late EBV genes is largely based on data obtained from studies of other, albeit related, herpesviruses. The exact molecular features of this process in the case of EBV remain to be determined.

In the context of the initiation of transcription of late EBV genes, it should also be noted that their transcription occurs only if the viral genome replication process is successful (Djavadian et al., 2018). Based on the fact that only newly synthesized viral DNA in the RC in the BMRF1-bound state does not contain histones and is unmethylated compared to the long-existing episomal form, some researchers suggest that BcRF1 and vPIC in general may have a sufficient level of affinity for this epigenetically distinct form of the viral genome (Chakravorty et al., 2019). This assumption may also be supported by the generally relatively low concentrations of vPIC components (Ersing et al., 2017). It is likely that it is within the RC that their concentration may be sufficient for successful transcription.

The structural proteins BcLF, BFRF3, BDLF1, BORF1, BGLF1, and BBRF1, tegument proteins, lipid envelope glycoproteins BALF4 (gB), BXLF2 (gH), BZLF2, BLLF1, and BILF2, as well as accessory factors BVRF2, BFRF0.5, BFLF1, BFRF1, and BXRF1 are dependent on vPIC and, accordingly, can be considered late ones (Dremel and Didychuk, 2023).

**Fig. 12:**
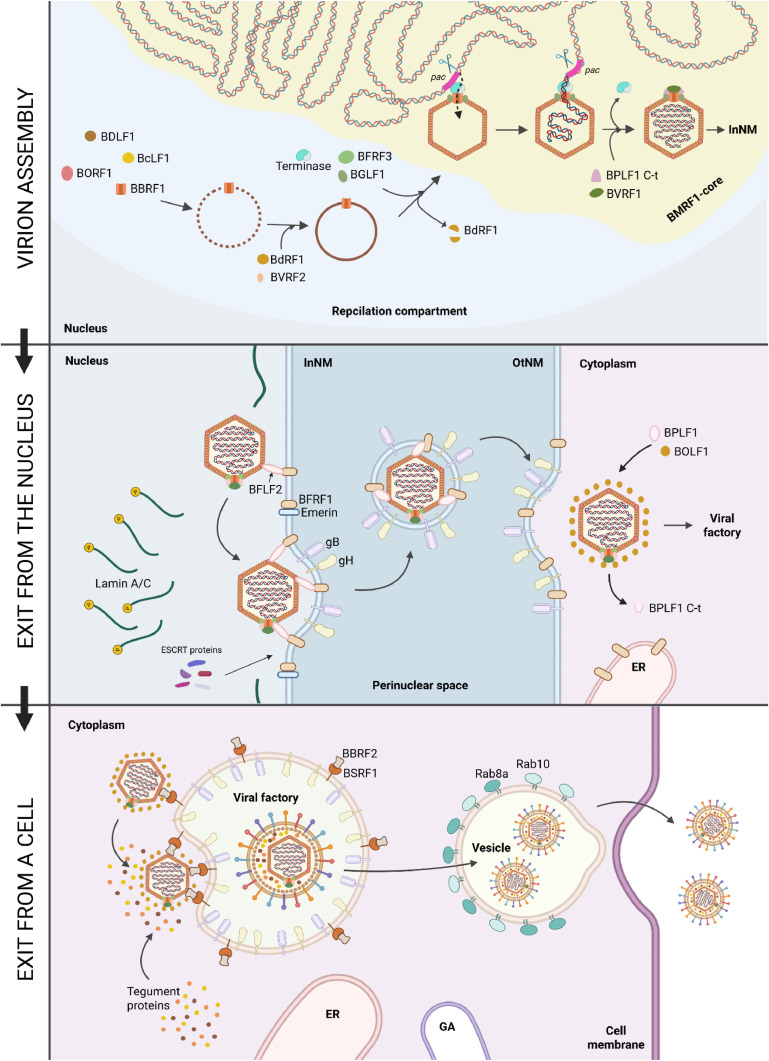
The scheme of the stages and processes occurring in the late stage of the lytic cycle of EBV. InNM - inner nuclear membrane; OtNM - outer nuclear membrane; ER - endoplasmic reticulum; GA - Golgi apparatus. Created in BioRender. Zaremba, P. (2025) https://BioRender.com/riremoe.

In general, despite the fact that among gammaherpesviruses, the assembly of the EBV virion is perhaps the best studied, the amount of data on the direct processes that occur at the molecular level during capsid formation, its maturation, and cellular transport is quite limited compared to, for example, HSV (Heming et al., 2017). However, modern studies of this stage of the EBV life cycle do not, for the most part, contradict the data obtained from studies of other members of the family (Möhl et al., 2016; Liu et al., 2020). Therefore, it makes sense to consider this process, similarly to many described above, including at the level of analogy (Fig. 12).

It is believed that EBV capsid assembly, similar to other herpesviruses, begins with the association of BBRF1 (portal), BcLF1 (mcp), BORF1 (tr1), and BDLF1 (tr2), where BBRF1 is considered to be the starting point for crystallization. This process occurs outside the BMRF1 core and leads to the formation of unstable conglomerates, which are stabilized by association with the scaffold protein BdRF1 and the protease BVRF2 (Sugimoto et al., 2019). Thus, the first early spherical form of the procapsid is formed (Tatman et al., 1994). For its angularization, the terminase complex must interact with the portal and induce BVRF2 activation. The mechanism of the latter is currently unknown. However, it is known that upon activation, this serine protease cleaves BdRF1 and thus leads to procapsid compaction (Heymann et al., 2003). The angular shape of the procapsid is stabilized by BFRF3, which binds to each BcLF1 within every hexon.

Based on the major concentration of BFRF3 within the BMRF1 core, as well as the similar positioning of the major EBV capsid localization factor protein BFLF1, angularization and subsequent maturation steps likely occur already in this subcompartment (Sugimoto et al., 2019). This same statement is confirmed by the direct detection of mature EBV capsids directly in the central zone of the BMRF1 conglomerate (Sugimoto et al., 2019). As we described above, this compartment contains the main array of concatemeric DNA. The presence of an empty capsid here allows for the packaging of the EBV genome with the least energy loss.

The actual packaging process is thought to be mediated by the aforementioned hexameric terminase complex, which is essentially a channel with a diameter of about 39 Å. Each monomer of the channel, in turn, is a heterotrimer of the proteins BGRF1/BDRF1, BALF3, and BFRF1A (Yang et al., 2020). Despite the fact that there is data that postulates a slightly different function of each protein, the close intertwining and functional dependence allows us to consider the trimer as a single structural-functional conglomerate (Yang et al., 2008). According to a recently developed theory based on structural data, each of the six nodes has ATPase, nuclease, and DNA-binding activities (Yang et al., 2020). The initial recognition of DNA is probably based on the sequence in the region of one of the *pac* sites. Located at the start position closer to the outer part of the hexameric ring, the active sites of the endonuclease cleave the concatemer, thus forming a free end, which is captured and positioned by the inner part of the terminase complex. Positioning is probably carried out in such a way that the DNA duplex interacts with the interior of two trimers at once, which for better understanding can be conditionally called S1 and S2. The zone of interaction with DNA of each trimer can be conditionally divided into two subzones, strongly related to DNA and weakly related one, which alternate within the terminase hexamer. In the initial position, the DNA duplex interacts with the weak binding site S1 and the strong binding site S2. Interaction with S1 triggers a series of conformational rearrangements that are transmitted to S2 and lead to the activation of its ATPase activity. Hydrolysis of ATP to ADP triggers a series of conformational rearrangements of S2, which are manifested in a shift of the bound DNA by 15° along the helix towards the capsid. This leads to a decrease in the affinity of S2 for DNA and an approach of the duplex to the strong binding site of conditional S3. Strong binding repositions the nucleic acid to a similar position to the original one, but with part of DNA already moved into the capsid, and S2 acts as a trimer, which provides activation of the ATPase activity of S3. These "pairwise" cycles are repeated, probably until the terminase reaches the second *pac* site, which is recognized by the endonuclease (DNA is cut again at this site). Although there is an assumption that the determination of the end of packaging occurs by transmitting tension by the capsid proteins through the dodecameric portal to the terminase complex, which leads to a change in the conformation of the trimers, in which the endonuclease active center shifts to the inside of the channel and hydrolyzes it (Wang et al., 2020). In general, these issues are still actively researched, even for the Herpesviridae family.

The virion assembly and packaging stage should be supplemented by another feature of herpesviruses: the presence of a capsid-associated tegument complex. In the case of EBV, it includes BGLF1 (UL17), two copies of BVRF1 (UL25) and two copies of BPLF1 (UL36). In the case of HSV, this heteropentamer interacts with one of the faces adjacent to the apex of the capsid. This applies to all vertices, including the one where the portal is located. Thus, a five-membered complex of CATC proteins is formed at each vertex (Wang et al., 2018). Although regarding EBV this heteropentamer is present in only ∼20 % of cases in the form of 1–2 copies (Liu et al., 2020). This is apparently due to the lower need of EBV for transport of virions in the early stages of infection compared to HSV. However, in both cases, the vertex with the portal is completely filled. This is explained by the importance of CATC proteins in DNA packaging, since in the absence of any of them, the capsids formed during infection do not contain nucleic acid (Möhl et al., 2016). Looking at the CATC components in more detail, BGLF1, through its interaction with triplex proteins, is likely responsible for the initial association of CATC with the capsid and serves as a base for the rest of the CATC. It may also be involved in signaling the completion of the capsid, as it is the only CATC required for the cleavage of concatemeric DNA (Huet et al., 2016; Stow, 2001). BVRF1 is likely required to counteract premature exit of the EBV genome from the virion, given its previously described role in injecting the viral genome into the nucleus. The requirement for BPLF1 during assembly is less clear. It is known that in the virion this protein attaches a complex of BGLF1 and BVRF1 to the penton, thus likely stabilizing the entire protein conglomerate (Dai et al., 2014). However, it is not known whether it is part of the CATC in the nucleus in its full or truncated form. The latter is based on the features of the electron microscopy density distribution of CATC in the nucleus for KSHV, which indicates the presence of a small protein there (Fan et al., 2015). Based on this, it is postulated that the CATC of KSHV, and possibly also HSV, is stabilized in the nucleus by the C-terminus of the BPLF1 homolog. Returning to EBV, the ability of BPLF1 to be a substrate for caspase-1 has been confirmed (Gastaldello et al., 2013). The N-terminal fragment is a highly active deubiquitinase capable of weakening the processes of polypeptide degradation stimulated by the massive production of viral proteins and significantly deregulate signaling cascades such as mTOR (Mund et al., 2025). Thus, it seems likely that during the formation of CATC in the nucleus, it is the C-terminal fragment of BPLF1 that is included in its composition. This logically complements viral reproduction with the idea of ​​the dependence of the number of mature nucleocapsids on the activity of cellular caspases as markers of approaching apoptosis.

The kinetics of association of each of these proteins with the capsid are currently poorly understood. Although it is known that BGLF1 and BVRF1 are mostly located within the BMRF1 core (Sugimoto et al., 2019). Given these data, as well as the functionality of the CATC components, a model in which BGLF1 associates with triplex proteins first, perhaps even before the onset of angularization, seems plausible. Then, after packaging is complete, BVRF1 associates with BGLF1 and, after dissociation of the terminase complex, closes the channel formed by the portal dodecamer. At the final stage, the C-terminal fragment of BPLF1 stabilizes CATC, and hence the capsid, by additional binding to pentons.

After nucleocapsid formation is complete, the next stages of the EBV life cycle occur outside the host cell nucleus. The EBV exit from the nucleus occurs according to the mechanism characteristic of herpesviruses "envelopment-deenvelopment-reenvelopment" and involves significant changes in the nuclear cytoskeleton and membranes, which begin with the aforementioned weakening of the nuclear lamina through the functioning of the BGLF4 kinase. The next proteins to be included are BFRF1 and BFLF2, which are components of the nuclear egress complex (NEC) (Lv et al., 2018). The first one is an integral membrane protein, which is mostly represented in the endoplasmic reticulum, inner and outer nuclear membranes, and the second one is a phosphoprotein of the nuclear matrix. (Lee and Chen, 2021). BFRF1, even when expressed independently, is capable of leading to significant reorganization of the nuclear membrane with simultaneous accumulation of cytoplasmic vesicles (Lee et al., 2012). This is likely related to the ability of this protein to recruit components of the endosomal sorting complex required for transport (ESCRT). ESCRT is normally responsible for the encapsulation and directed transport of ubiquitinylated cell surface proteins, and is also involved to some extent in most processes associated with cell membrane remodeling (Hurley, 2010). Furthermore, BFRF1 is able to interact with emerin, which is a lamin A/C receptor localized to the inner nuclear membrane. This likely leads to an enfeebling of the connection of the already weakened nuclear lamina to the nuclear envelope (Leach et al., 2007). Thus, this membrane protein ensures that EBV rearranges the nuclear membrane in a manner necessary for nuclear export. Another NEC protein, BFLF2, has been studied much less thoroughly. However, its removal is known to lead to the accumulation of abnormal numbers of immature EBV capsids in the nucleus, suggesting a yet unknown function of this protein in nucleic acid packaging (Granato et al., 2008). It is also clear that BFLF2 interacts with BFRF1 via a fairly powerful interaction interface (Dai et al., 2020). Thus, the adaptor function of this viral protein seems logical. BFLF2, due to at least double affinity, is able to recruit EBV nucleocapsids to the inner nuclear membrane, modified by BFRF1 and the ESCRT factors recruited by the latter.

After the formation of the primary vacuole, which contains one or more EBV nucleocapsids and is located in the periplasmic space, it fuses with the outer nuclear membrane by a largely unknown mechanism. Thus, its contents are released into the cytoplasm. Based on homologies, it can be assumed that in addition to BFRF1, which is also present on the outer nuclear membrane, surface gB and gH may also participate in this process (Farnsworth et al., 2007).

The subsequent processes of tegumentation and virion release from the infected cell, unfortunately, have been studied very superficially in the case of EBV. However, the currently available limited experimental data do not contradict current studies conducted on other herpesviruses (Li et al., 2020; He et al., 2020).

In the cytoplasm, the nucleocapsid first replaces the truncated form of BPLF1 with the full-length BPLF1. This results in the formation of the first complete layer of the icosahedral inner tegument. The second layer is probably formed by the BOLF1 protein (Mettenleiter, 2002). During the same period, the remaining tegument proteins accumulated since the beginning of the infection are mostly located in one area of ​​the cytoplasm due to the complex cross-interaction patterns existing between them. The area is similar to a specific membrane structure of alphaherpesviruses (Nanbo et al., 2018). Although it seems that in the case of EBV, in addition to the cis-Golgi and trans-Golgi, macroautophagy mechanisms are also involved in the formation of this membrane substructure (Nowag et al., 2014). Thus, the process of formation of the outer tegument and the actual acquisition of the final lipid membrane by the EBV virion takes place within the virus-specific cellular compartment, essentially a cytoplasmic factory, which contains both tegument proteins and lipid vesicles with EBV terminal glycoproteins processed in the Golgi apparatus.

At this stage, the role of the proteins BBRF2 and BSRF1 has been proven. (He et al., 2020). BSRF1 is a tegument protein that is palmitated at a conserved among herpesviruses cysteine ​​residue at the N-terminus (Nozawa et al., 2003). This provides the affinity of this protein to lipid membranes, in particular to derivatives of the Golgi apparatus. At the same time, BSRF1 has an affinity for BBRF2, thus attracting this protein to the membrane surface. BBRF2, in turn, has an affinity for BPLF1 and BcLF1. Acting, in essence, as a bridge between the nucleocapsid enveloped by the inner tegument and BSRF1 anchored in the membranes of the cytoplasmic factory. It is assumed that the BSRF1/BBRF2 complex, which also demonstrates a tendency to oligomerize, lines the cytoplasmic side of the above-mentioned membranes, which allows directing nucleocapsids to the site of final maturation (He et al., 2020).

After the initial interaction with the future membrane, the attachment of the outer tegument proteins is initiated. The latter form an amorphous mass located in the future virion mostly on the distal side relative to the nucleocapsid (Tsai et al., 2013). Further, by a currently unexplored mechanism, such a tegumented nucleocapsid buds into the vesicular lumen, thus forming a mature EBV virion with a nucleocapsid slightly displaced from the center.

Cytoplasmic factory vesicles containing sufficient numbers of formed EBV virions are transported to the plasmalemma, probably exploiting cellular exocytosis mechanisms (Nanbo, 2020). This statement is based on the observations of colocalization of the GTPases Rab8a, Rab10, and even Rab11a with gp350/220, as well as the apparent inhibition of EBV virion release when these factors are knocked out. Although the reason for the involvement of these key vesicular transport factors is actually unknown. As is the pattern of regulators involved in this case, which are known to determine the functional activity of Rab GTPases (Homma et al., 2021).

The final stage of the life cycle of any virus is its exit from the host cell. In the case of EBV, this occurs through the fusion of the membrane of the vesicle carrying virions and plasmalemma. However, the molecular mechanisms of this process are unknown not only for EBV, but also for the entire Herpesviridae family (Tognarelli et al., 2021). This is obviously a consequence of the lack of reliable approaches to studying the kinetics of such processes. In this context, the above-mentioned BALF0/1 should also be brought up. In addition to its anti-apoptotic function, Yiu et al. documented the ability of BALF0/1 to enhance BCR degradation through ubiquitin-mediated proteasomal pathway (Yiu et al., 2025). Thus, the authors believe that EBV prevents the unwanted association of newly formed virions with the “mother” cell, which contains BCRs targeting its surface antigens. This study also indirectly confirms the above-mentioned assumption that an EBV-infected B-lymphocyte undergoes affinity maturation specifically for EBV antigens during the establishment of its latency.

## 12 Conclusion

This work is a structured summary of the global progress of more than sixty years of research on the Epstein-Barr virus. Within the framework of the sequential coverage of each stage of the EBV life cycle, we reveal the current understanding of the complex progressive relationships between the virus, the infected cell and the host immune system. When writing this review, we sought to provide a reader with a complete picture of the life cycle of this very common pathogen, which, at the same time, is associated with a number of serious diseases.

An additional consequence of the processing of such an array of information was the understanding of the white spots present in the spectrum of research on this virus. Along with many of the areas of reduced interest of the scientific community mentioned and highlighted in our work, we would recommend that researchers pay special attention to the functions of each of the tegument proteins (especially the outer ones) and their specific role in the processes of nucleocapsid transport, virion assembly and control of cellular metabolism both at the beginning and at the end of infection. There is also a clear lack of data on morphological, morphometabolic and morphogenetic studies of EBV-infected cells in the context of the affinity maturation process. Overall, we would characterize the level of research on the Epstein-Barr virus life cycle as requiring further large-scale multidisciplinary studies.

## Funding declaration

This research received no funding.

## CRediT authorship contribution statement

**Andrii Zaremba:** Conceptualization, Data curation, Formal analysis, Investigation, Validation, Project administration, Writing – original draft. **Polina Zaremba:** Visualization, Writing – review & editing. **Svіtlana Zahorodnia:** Supervision.

## Declaration of competing interest

The authors declare that they have no known competing financial interests or personal relationships that could have appeared to influence the work reported in this paper.
